# Alternative Wnt-signaling axis leads to a break of oncogene-induced senescence

**DOI:** 10.1038/s41419-024-06550-8

**Published:** 2024-02-22

**Authors:** Viola Kluge, Melanie Kappelmann-Fenzl, Stefan Fischer, Tom Zimmermann, Michaela Pommer, Silke Kuphal, Anja-Katrin Bosserhoff

**Affiliations:** 1 Institute of Biochemistry, Friedrich-Alexander-Universität Erlangen-Nürnberg (FAU), Erlangen, Germany; 2 Faculty of Computer Science, Deggendorf Institute of Technology, Dieter-Görlitz-Platz 1, 94469 Deggendorf, Germany

**Keywords:** Melanoma, Tumour heterogeneity, Cell growth

## Abstract

Oncogene-induced senescence (OIS) is an important process that suppresses tumor development, but the molecular mechanisms of OIS are still under investigation. It is known that BRAF^V600E^-mutated melanocytes can overcome OIS and develop melanoma, but the underlying mechanism is largely unknown. Using an established OIS model of primary melanocytes transduced with BRAF^V600E^, YAP activity was shown to be induced in OIS as well as in melanoma cells compared to that in normal epidermal melanocytes. This led to the assumption that YAP activation itself is not a factor involved in the disruption of OIS. However, its role and interaction partners potentially change. As Wnt molecules are known to be important in melanoma progression, these molecules were the focus of subsequent studies. Interestingly, activation of Wnt signaling using AMBMP resulted in a disruption of OIS in BRAF^V600E^-transduced melanocytes. Furthermore, depletion of Wnt6, Wnt10b or β-catenin expression in melanoma cells resulted in the induction of senescence. Given that melanoma cells do not exhibit canonical Wnt/β-catenin activity, alternative β-catenin signaling pathways may disrupt OIS. Here, we discovered that β-catenin is an interaction partner of YAP on DNA in melanoma cells. Furthermore, the β-catenin–YAP interaction changed the gene expression pattern from senescence-stabilizing genes to tumor-supportive genes. This switch is caused by transcriptional coactivation via the LEF1/TEAD interaction. The target genes with binding sites for LEF1 and TEAD are involved in rRNA processing and are associated with poor prognosis in melanoma patients. This study revealed that an alternative YAP-Wnt signaling axis is an essential molecular mechanism leading to OIS disruption in melanocytes.

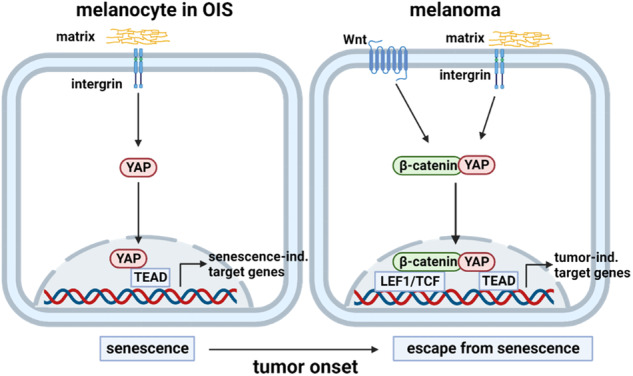

## Introduction

Cutaneous melanoma arises by the malignant transformation of melanocytes, which are derived from the neural crest and are located in the basal layer of the epidermis. The incidence of malignant melanoma is still steadily increasing, and it is characterized by a high rate of metastasis. Despite major advances in targeted therapy and immunotherapy in recent years, the late stages of this disease are still not fully cured [1]. Therefore, understanding the underlying molecular mechanisms of pathogenesis is essential for developing new therapeutic treatment strategies and methods for early diagnosis.

Cellular senescence, first described by Hayflick and Moorhead in 1961, describes a stable state of growth arrest and has been linked to numerous physiological and pathological conditions [2]. The process of senescence is caused by different mechanisms, such as telomere shortening, DNA damage and oncogene activation [3]. In primary melanocytes, the oncogenic activation of BRAF^V600E^ (BRAFm) induces cell cycle arrest, which is considered to be a tumor suppressive mechanism and results in the development of benign nevi [4]. In previous studies, we showed that molecules such as melanoma inhibitory activity (MIA) and amphiregulin (AREG) are important factors for maintaining senescence in melanocytes [5–8]. Senescent cells remain metabolically active but arrest at the G_0_/G_1_ phase due to the induction of tumor suppressors, including p16^INK4A^ and p21 [4, 9]. The senescent phenotype of cells is highly heterogeneous, resulting in the use of many markers to analyze senescence. The molecular regulation of the development of melanoma derived from nevi and senescent melanocytes is largely unknown, although understanding this process is highly important.

Deregulation of the Hippo pathway is one event contributing to cancer development and leading to a kinase cascade in which macrophage stimulating 1/2 kinases (MST1/2) phosphorylate large tumor suppressor kinase 1/2 (LATS1/2) and Salvador family WW domain containing protein 1 (SAV1). LATS1/2 kinase in turn phosphorylates and inhibits the transcriptional coactivators yes associated protein/transcriptional coactivator with PDZ-binding motif-protein (YAP/TAZ), two major downstream effectors of the Hippo pathway. In the inactive state of the Hippo pathway, YAP/TAZ are dephosphorylated, translocated into the nucleus and form DNA-binding complexes with TEA domain transcription factor 1–4 (TEAD1-4) and other transcription factors. This leads to the stimulation of gene expression, resulting in the regulation of cell proliferation, apoptosis, and stem cell renewal [10].

Another pathway known to play an important role in melanoma is the Wnt signaling pathway, which includes noncanonical and canonical pathways. The noncanonical Wnt pathway is independent of β-catenin. A typical example of a β-catenin-independent pathway is the Wnt/Ca^2+^ pathway and the noncanonical Wnt planar cell polarity signaling pathway [11]. In the absence of Wnts, the transmembrane receptors frizzled class receptor (FZD) and LDL receptor-related protein 5/6 (LRP5/6) are located separately on the plasma membrane. In the canonical pathway, β-catenin binds to a “destruction complex” comprising adenomatous polyposis coli (APC), AXIN, casein kinase 1 (CK1) and glycogen synthase kinase 3 protein (GSK3) in the cytoplasm and captures β-catenin by phosphorylating [12]. Binding of Wnts to their receptors leads to disruption of the “destruction complex”, and free β-catenin translocates to the nucleus where it functions as a transcriptional coactivator; for example, of the transcription factor/lymphoid enhancer binding factor 1 (TCF/LEF1) DNA-binding transcription factor, and stimulates gene expression [12, 13]. Wnt signaling is often implicated in stem cell control as a proliferative and self-renewal signal [14–16]. The expression of specific Wnts has been described not only during development, growth and proliferation but also in different diseases and tumor types, especially during tumor onset and early stages [17–19]. For example, Wnt1 and Wnt3a promote melanocyte differentiation and melanoma development via β-catenin-dependent signaling. Wnt5a is another important ligand that increases the invasiveness of melanoma cells [20–22]. It has also been reported that 30% of melanoma biopsies possess β-catenin in their nuclei, suggesting that the Wnt/β-catenin pathway might be activated in malignant melanoma [23]. To determine the specific role of β-catenin in vivo, the Larue laboratory generated transgenic mice expressing an activated form of β-catenin in melanocytes. Interestingly, in their model, the authors showed that active β-catenin is not sufficient for melanoma development but results in the immortalization of melanocytes [24]. The exact role of the hippo pathway and the β-catenin pathway has not been defined yet in melanoma development and progression, although an interaction between both pathways has been described in different cell types [25–27]. In melanoma-associated fibroblasts a YAP-β-catenin signaling axis was confirmed, which increased the tumor promoting function of fibroblasts [27]. In this study, we revealed that Wnt ligands are differentially regulated in melanoma cells compared to melanocytes and that upregulation of Wnt ligands and activation of β-catenin leads to a disruption of OIS in melanocytes. Further, we present a so far unknown Wnt-YAP signaling axis with an interaction of YAP and β-catenin in melanoma cells. Inhibition of this signaling axis in melanoma cells leads to a re-induction of senescence in melanoma cell lines. Our findings propose that the alternative Wnt-YAP signaling axis plays an important role in overcoming oncogene-induced senescence in melanocytes and promotes tumor development.

## Materials and methods

### Cell culture

As described previously [28] normal human melanocytes (NHEM, neonatal) were obtained from Lonza and cultivated in MGM-4 BulletKit medium (Lonza, Basel, Switzerland) with 1% penicillin/streptomycin. NHEM from different donors were used between passages 6 and 8. HEK293T (ATCC-CRL-3216) cells were a generous gift of Prof. Stephan Hahn (RuhrUniversität Bochum, Germany). Cells were cultivated in high-glucose Dulbecco’s modified Eagle’s medium (DMEM) supplemented with penicillin (400 U/mL), streptomycin (50 µg/mL), and 10% fetal calf serum (all from Sigma-Aldrich, Munich, Germany). NHEM and HEK293T cells were incubated at 37 °C and 5% CO_2_ in a humidified atmosphere. Human melanoma cell lines SBcl2 (RRID: CVCL_D732), WM1366 (RRID: CVCL_6789) (a generous gift from Dr. M. Herlyn, Wistar Institute, Philadelphia, USA) derived from RGP (radial growth phase) (SBcl2) or VGP (vertical growth phase) (WM1366) melanoma were maintained in a culture medium consisting of MCDB153 (Sigma-Aldrich, Steinheim, Germany) with 20% Leibovitz’s L-15 (PAA Laboratories, Coelbe, Germany), 2% FCS, 1.68 mM CaCl_2_ (Sigma), and 5 µg/ml insulin (Sigma-Aldrich, Steinheim, Germany) at 37 °C and 5% CO_2_ [29]. RGP includes melanoma in situ, has no metastatic potential and a good prognosis whereas VGP is associated with higher metastatic potential and a poor prognosis. Human melanoma cell lines MV3 (RRID: CVCL_W280) (derived from metastases of malignant melanoma) were cultured in high-glucose Dulbecco’s modified Eagle’s medium (DMEM) supplemented with penicillin (400 U/ml), streptomycin (50 μg/ml) and 10% fetal calf serum (all from Sigma-Aldrich, Munich, Germany). Human melanoma cell lines A-375 (RRID: CVCL_0132) (derived from primary tumor) and Mel Im (RRID: CVCL_3980) (derived from metastases of malignant melanoma) were cultured in low-glucose Dulbecco’s modified Eagle’s medium (DMEM) supplemented with penicillin (400 U/ml), streptomycin (50 μg/ml) and 10% fetal calf serum (all from Sigma-Aldrich, Munich, Germany). Mycoplasma contamination was regularly excluded for all primary cells and cell lines. The authors confirm the authentication of all cell lines used in the research.

### SiRNA and plasmid transfection

The melanoma cell lines SBcl2, WM1366 and MV3 were transfected with siRNA pools against YAP, β-catenin (CTNNB1), TEAD, Wnt6, Wnt10b, TCF7, TCF7L2 and LEF1 (siTools Biotech GmbH, Planegg, Germany [30]) and a siCtr, respectively, using the Lipofectamine RNAiMAX reagent (Life Technologies, Darmstadt, Germany), as described previously [31]. Depending on the cell line, 200 000 cells (WM1366, MV3) or 400 000 cells (SBcl2) were seeded in 6-well plates for 48 h transfection. 50 pmol of siRNA was added to cells. Plasmid transfections were performed using 0.5 µg vector DNA and LTX plus Lipofectamine for 24 h or 48 h transfection time.

### Lentiviral transduction of melanocytes

Lentiviral transduction using a third-generation vector system was described elsewhere [28]. At a density of 2 × 10^6^ cells/plate, HEK293T cells were seeded in 10 cm plates. The next day, three vectors, an envelope plasmid pHIT-G, a packaging plasmid pCMV ΔR8.2 and a target plasmid with the DNA of interest (either copGFP, B-Raf^V600E^, mcherry Ctr or mcherry β-cateninS33Y) were transfected using transfection with Lipofectamine^®^ LTX (Thermo Fisher, Waltham, MA, USA). Cells were incubated 16 h before the medium was changed to MGM-4 BulletKit medium. After an incubation for 24 h, supernatants were collected, filtered and applied to NHEM. Polybrene^®^ (Santa Cruz, Dallas, TX, USA) was added to a final concentration of 1 µg/ml. For viral transfection with two plasmids (mock/BRAFm and mcherry β-cateninS33Y/mcherry Ctr) supernatant of both plasmids was added at the same time to NHEM. Lentiviral supernatants were removed after 16 h. Cells were washed three times with PBS and cultivated in regular MGM-4 BulletKit medium. All experiments using transduced melanocytes started exactly 7 days after transduction to allow establishment of a senescent phenotype.

### Co-culture

Four days after viral transfection, NHEMs were seeded in 24-well plates. At the same time, 10^6^ HEK293T cells were seeded in 6-well plates and after 2 h transfected with Wnt6 or CMV overexpression plasmid as described in “siRNA and plasmid transfection”. Active Wnt6-V5 was a gift from Xi He (Addgene plasmid # 43815; http://n2t.net/addgene:43815; RRID:Addgene_43815) [32]. 10 000 HEK93T cells were then seeded in inserts after 4 h incubation and added in a 24-well plate with transduced NHEM. NHEMs and HEK293T cells were co-cultured for 72 h in MGM-4 BulletKit medium.

### Agonist and recombinant protein

Seven days after viral transfection, NHEMs were treated with recombinant Wnt10b (200 ng/ml**;** R&D Systems, Inc., Minneapolis USA) with PBS as control and AMBMP hydrochloride (50 nM; R&D Systems) with DMSO as control.

### Cloning

β-cateninS33Y was cloned into lentivirus pLV-mcherry plasmid. pLV-mCherry was a gift from Pantelis Tsoulfas (Addgene plasmid #36084; http://n2t.net/addgene:36084; RRID:Addgene_36084). This β-catenin, containing a missense mutation of tyrosine for serine at codon 33 (S33Y), was isolated from the human colon cancer cell line SW48 and is constitutively active [33]. β-cateninS33Y sequence was amplified from pcDNA3-S33Y Beta-catenin, which was a gift from Eric Fearon (Addgene plasmid #19286; http://n2t.net/addgene:19286; RRID:Addgene_19286), with specific forward and reverse primers from Sigma-Aldrich containing BamHI and XbaI restriction sites [34]. PCR Cloning Kit “2.0” (New England Biolabs ® Inc., Ipswhich, MA, USA) was used.

### Analysis of mRNA expression using real-time PCR

Isolation of total cellular RNA and generation of cDNAs by reverse transcription reaction was performed as described previously. Quantitative real-time PCR (qRT-PCR) analysis of gene expression was performed on a LightCycler 480 system as described elsewhere [35]. Specific set of Primer sequences are in Table 1.

**Table 1:** Primer used for quantitative real-time PCR.

Gene	Forward Primer	Reverse Primer
AREG	TCGGCTCAGGCCATTATGCT	CTCCCGAGGACGGTTCACTA
AXL	GACATCCTCTTTCTCCTGCGA	CACATCGCTCTTGCTGGTGTAGACACGGT
CTGF	CAGAACCACCACCCTGCCG	CGTACATCTTCCTGTAGTACA
LEF1	TGCAGCTTTATCCAGGCT	GTCTCTAGCAGTGACCTCAGG
TCF7	CCAAGAATCCACCACAGGAGG	CGCAGGGCTAGTAAGCAGTT
TCF7L2	CAAGAGGCAAGATGGAGGGC	GTAATGTGTGCTGCCGGACT
Wnt10b	TTGGAGGGTCTTGAGGGGAA	TCCCTCAAGGGAGAGGAGTG
Wnt10b	TCCACTGGTGCTGCTATGTG	ACACATCCCAGAGTCCACCT
Wnt10b	TCCTCAAGCGCGGTTTCC	AACTCTTGCCTCGGGACAG
Wnt2	TATCAGGGACCGAGAGGCAG	GGTTGTCCAGTCAGCGTTCT
Wnt3	TGACTCGCATCATAAGGGGC	GCCTCGTTGTTGTGCTTGTT
Wnt5a	AGGGCTCCTACGAGAGTGCT	GACACCCCATGGCACTTG
Wnt5b	CCTGTCTTTGGCTCGGAAAC	TAATGACCACCAGGAGTTGGC
Wnt6	CAGCTCGAAGAGAACTGCCT	GCCTCACCATTTCCAGAGCC
Wnt6	GCAGCCCCTTGGTTATGGAC	CGTCTCCCGAATGTCCTGTT
Wnt9a	AGCGCGATGGTCGGC	CCCGTCAGCCCGAAGTAG
YAP	CCCTCGTTTTGCCATGAACC	ACCATCCTGCTCCAGTGTTG
β-actin	CTACGTCGCCCTGGACTTCGAGC	GATGGAGCCGCCGATCCACACGG
β-catenin	TTTGATGGAGTTGGACATGGC	TGATGGTTCAGCCAAACGC

### Western blot protein analysis

Cell pellets were lysed in 50 µl RIPA buffer (Roche, Mannheim, Germany) for 20 min at 4 °C. Cell fragments were removed by centrifugation (13 000 rpm, 10 min, 4 C), and the supernatant was collected. Western blot analysis was performed as described previously [36]. 20–40 µg protein were loaded on SDS polyacrylamide gels for electrophoresis and blotted onto a PVDF membrane (Bio-Rad, Hercules, CA, USA). Membranes were blocked for 1 h using 5% non-fat dried milk/TBS-T or 5% BSA. Primary antibodies against YAP (1:1 000 in 5% BSA, Cell Signaling, D8H1x), TEAD (1:1 000 in 5% BSA, Cell Signaling, D3F7L) or β-catenin (1:4 000 5% BSA, Sigma-Aldrich) were incubated overnight at 4 °C. The primary antibody against β-actin (1:2 000 in TBS-T, Sigma Aldrich, A5441) was incubated for 1 h at room temperature. Secondary antibodies conjugated to horseradish peroxidase (HRP, Cell Signaling, 7074 and 7076) were applied for 1 h at room temperature. For visualization, Clarity™ Western ECL Substrate (Bio-Rad) was added to the membranes and imaged by Chemostar chemiluminescence imager (Intas, Goettingen, Germany). The densitometry was performed using LabImage software (Kapelan Bio-Imaging GmbH, Germany).

### Co-immunoprecipitation

As described previously [37], 3 × 10^6^ MV3, SBcl2 or WM1366 cells were lysed in 100 µL of RIPA buffer (Roche) and incubated for 20 min at 4 °C. Cell fragments were removed by centrifugation (13,000 rpm for 10 min at 4 °C), and the supernatant was stored at −20 °C. Protein-G-sepharose beads (GE Healthcare, Munich, Germany) were rinsed three times with ice-cold PBS (used in all subsequent washing steps) and incubated with 250 µg of protein at 4 °C for 4 h. The beads were removed by centrifugation at 3 000 rpm for 1 min at 4 °C. Precleared proteins were incubated with anti-YAP, anti-β-catenin and anti-IgG (Cell Signaling, DA1E) primary antibodies at 4 °C overnight. 20 µg washed Protein-G-sepharose beads were incubated with protein-antibody mix at 4 °C overnight. The beads were washed four times with ice-cold PBS and resuspended in 30 µL of Roti®Load (Carl Roth GmbH + Co. KG, Karlsruhe, Germany) and heated at 95 °C for 5 min. The whole supernatant was loaded onto SDS polyacrylamide gels for electrophoresis as described before [38].

### Immunofluorescent staining

As described previously [39], 20 000 cells were seeded on 18 mm round coverslips. The next day, cells were washed twice with PBS and fixed with 2% paraformaldehyde for 12 min and washed three times with PBS. Permeabilization using 0.1% Triton-X100 in PBS for 3 min was followed by 30 min blocking with 10% BSA in PBS. The primary antibody against PML (1:200 in 1.5% BSA/PBS, Santa Cruz, sc-966), KI-67 (1:500 in 1.5% BSA/PBS, Abcam, ab16667), YAP (1:100 in 1.5% BSA/PBS, Cell signaling, D8H1x) was added and incubated overnight at 4 °C. The according secondary antibody Alexa 488, Alexa 555 or Alexa 647 (1:400 in 1.5% BSA/PBS, Thermo Fisher, A32727) was incubated for 1 h at room temperature. Cells were stained with DAPI (1:10 000 in PBS, Sigma Aldrich) 30 min and mounted on microscope slides using Aqua-Poly/Mount (Polysciences, Warrington, PA, USA).

For reporter analysis cells were transfected with YAP localization plasmid (pLVX-AcGFP-YAP1-2a-P2A-MBD-tdTomato-C1, kindly provided by Ingo Thievessen, Department Biophysics, FAU, Germany). After 48 h cells were fixed and stained with DAPI as described before. Final stainings were analyzed using an Olympus IX83 inverted microscope in combination with Olympus CellSens Dimension software (Olympus, Tokio, Japan).

### Immunohistochemical analysis

Human metastatic melanoma tissues were screened for Wnt6 and Wnt10b protein expression by immunohistochemistry. Sampling and handling of patient material was carried out in accordance with the ethical principles of the Declaration of Helsinki. The use of human tissue material had been approved by the local ethics committee of the University of Regensburg (application numbers 09/11 and 03/151). The samples were prepared as described previously [40]. Standard 5 μm sections of formalin‐fixed and paraffin‐embedded tissue blocks were used for immunohistochemical analysis of human tissue samples (tissue microarray (TMA) comprising specimens from benign nevi, primary melanoma and melanoma metastases) [41]. Immunohistochemical staining were performed using anti-Wnt6 antibody (Abcam, Cambridge, UK; ab50030 1:100) or anti-Wnt10b antibody (Abcam, Cambridge, UK; ab70816 1:100).

### Real-time cell proliferation analysis (RTCA) by xCELLigence system

The xCELLigence system (Roche, Mannheim, Germany) is based on measurement of electrical impedance and permits real-time analysis of migration and proliferation [35]. E-plates were used and basic protocols recommended by the manufacturer were followed [35]. Approximately 5000 cells/well were seeded and loaded into the device. Proliferation was monitored for 7 days. The parameter *slope* was normalized to control treatment.

### XTT cell viability assay

Exactly 5 000 cells/well were seeded in each well of a 96-well plate 7 days after transduction and were treated as described in “Agonist and recombinant protein”. Cell viability was assessed after an incubation period of 7 days (NHEM) using the Cell Proliferation Kit II (Roche) according to the manufacturer’s instructions [35]. A Clariostar Plus Multiplate reader (BMG Labtech, Ortenberg, Germany) was used for photometric detection.

### Staining of senescence-associated beta-galactosidase activity

Quantification of β-Galactosidase activity was performed depending on the assay, either 13 days post-transduction in NHEM or 48 h post-treatment in SBcl2, MV3 or WM1366. Fixation and staining were performed using the Senescence β-Galactosidase Staining Kit (Cell Signaling, Danvers, MA, USA) following the manufacturer’s instructions and as described previously [39]. An Olympus IX83 inverted microscope in combination with Olympus CellSens Dimension Software (Olympus) was used to acquire images of the staining, which were then quantified manually using ImageJ.

### Flow cytometry

For cell cycle analysis, melanoma cells were transfected with YAP, β-catenin, Wnt6, Wnt10b, or Ctr siRNA pools. Cell cycle analysis was performed using flow cytometry as described before [42]. Cells were fixed in 70% ice-cold methanol and washed two times with 0.2% BSA/PBS. RNA was degraded by digestion with 10 µg/µl RNAse A (Sigma) at 37 °C for 15 min. Cellular DNA was stained with 25 µg/ml propidium iodide (PromoCell GmbH, Heidelberg, Germany). The percentage of cells in G1, S, and G2 phase was analyzed by flow cytometry with a BD LSRFortessaTM X-20 instrument (BD Biosciences) and flow cytometry data were analyzed using FlowJo Software (Becton Dickinson, Ashland, OR, USA).

For reporter gene analysis, cells were seeded into a six-well plate and transfected with 0.5 µg of reporter constructs using LTX plus Lipofectamine after 24 h. The reporter construct pGL4.23 + MCAT Modul + eGFP contains four replicates of the TEAD consensus DNA-binding sequences, kindly given from AG Brabletz (Experimental Medicine, FAU Erlangen-Nürnberg). To determine transfection efficiency, the data were normalized to CMV-GFP. Therefore, cells were transfected with pcDNA3.1 + CMV-eGFP plasmid. Cells were suspended in 1% BSA/PBS and analyzed by flow cytometry described above. GFP-fluorescence, which is under the control of a CMV promotor, was set as 100%. In experiments with siRNA pool, GFP-fluorescence, which is controlled by YAP/TEAD activity, was divided into low and high staining corresponding to low or high YAP active cells.

### Reporter gene (Luciferase) assay

Approximately 200,000 WM1366 or MV3 cells/well, or 400,000 SBcl2 cells/well were seeded into 6‐well plates and transfected, as mentioned earlier. After 24 h, cells were transiently transfected with 0.5 μg of plasmid DNA. To analyze canonical β-catenin activity, we used TOPflash/FOPflash plasmids (Upstate, Lake Placid, NY, USA). pGL4.23 + MCAT Modul contains four replicates of the TEAD consensus DNA-binding sequences, which are mutated in pGL4.23 + MCAT Modul mut. These plasmids were a kind gift from AG Brabletz (Experimental Medicine, FAU Erlangen-Nürnberg). The firefly-luciferase reporter constructs pGL4.27 5xPAX3BS and PGL4.27 were provided by J. A. Epstein (Department of Cell and Developmental Biology) [43]. The TBX5 luciferase *cis*-element reporter was provided by E. Olson [44]. The cells were lysed 24 h after transfection, and the luciferase activity was quantified by a luminometric assay (Promega Corp., Madison, WI, USA). To determine transfection efficiency, the data were normalized to renilla luciferase activity. Therefore, cells were co‐transfected with 0.1 μg of the pRL‐TK plasmid (Promega).

### EMSA

A double stranded oligomeric binding site for TEAD (hMCAT 1xGT -5’- TTC GAT ACA CTT GTG GAA TGT GTT TGA TTT GTT AGC CCC G-3’ (sense) and hMCAT 1xGT -5’- CGG GGC TAA CAA ATC AAA CAC ATT CCA CAA GTG TAT CGA A-3’ (antisense) for TEAD consensus and hMCAT 1xGT mut-5’-TTC GAC ACT TGT GTG CGG TGT TTG ATT TGT TAG CCC CG-3’ (sense) and hMCAT 1xGT mut -5’- CGG GGC TAA CAA ATC AAA CAC CGC ACA CAA GTG TCG AA-3’ (antisense) for TEAD mutated consensus) (Sigma) was phospho-labeled. Exactly 5 µg nuclear protein extract was incubated in a 20 µl reaction mixture containing 10 mM HEPES pH 8, 50 mM NaCl, 0.1 mM EDTA, 5 mM MgCl2, 2 µg poly dG:dC (Invivogen, San Diego, USA), 2 mM DTT, 4 µg BSA and 5% glycerol for 10 min at room temperature. For supershift, 1 µl anti-YAP, anti-β-catenin, anti-YAP/β-catenin, anti-TEAD1-4 or IgG control (Sigma) was added to the nuclear extract. Oligonucleotide probe was added and incubated 10 min at room temperature. DNA protein complexes were separated with 4.8% DNA retardation gels in TBE and transferred to Whatman filter paper (Carl Roth GmbH + Co. KG, Karlsruhe, Deutschland). The gels were dried at 75 °C for 105 min and exposed to autoradiographic analysis.

### cDNA array (Affymetrix)

Screening for differences in RNA expression level between mock NHEM and BRAF^V600E^ NHEM (*n* = 3), transduced as described in “Lentiviral transduction of melanocytes”, was performed using cDNA-array systems (Human Gene 2.0 ST Array*)*. Analyses were performed in cooperation with Dr. Thomas Stempfl at the KFB (Kompetenzzentrum Fluoreszente Bioanalystik, University Regensburg). Expression data are in the supplemental data (suppl Table 2).

### RNA-Sequencing and differential expression analysis

The total RNA samples were isolated using Total RNA kit I (Omega Bio-Tek, Inc., Norcross, GA, USA) according to manufacturer’s instructions. All RNA samples were examined for integrity and purity by the TapeStation 4200 (Agilent).

Library preparation was performed with at least two biological replicates using the TruSeq® Stranded Total RNA Library Prep Human/Mouse/Rat Kit according to the manufacturer’s instructions (Illumina Inc., San Diego, CA, USA). Sequencing was performed according to the paired-end RNA sequencing protocols from Illumina on a HiSeq4000 with a paired-end module (Illumina, Inc., San Diego, CA, USA). The samples were sequenced from each side of a fragment ~75 bp long with an average number of 20 million reads per sample. After quality check using FastQC (Babraham Bioinformatics—FastQC A Quality Control Tool for High Throughput Sequence Data), paired-end reads were aligned to the human reference genome (GRCh38.p5, release 24) using the STAR alignment software (v 2.7.9a) [45]. After mapping, only reads that mapped to a single unique location were considered for further analysis. The mapped reads were then used to generate a count table using the feature-counts software (v2.0.1) [Liao, Y.; Smyth, G.K.; Shi, W. featureCounts: An efficient general purpose program for assigning sequence reads to genomic features. Bioinformatics 2014, 30, 923–930.]. The raw reads were filtered, normalized, and visualized by using R (v4.0.5, The R Foundation for Statistical Computing). DESeq2 package (v1.28.1) [46] was used for logarithmic transformation of the data and for data exploration. PCA data were generated from the transformed results with DESeq2 and plotted using ggplot2 package version 3.3.5. Differential expression analysis was done using the DESeq2 standard approach. Adjusted *p*-values are calculated using the Benjamini–Hochberg method within DESeq2.Gene annotations were added to the result files using Ensemble data. Differentially expressed genes with an adjusted *p*-value < 0.1 were regarded as statistically significant.

### Finding motif instances across the whole genome

For motif site prediction we used the HOMER software tool [47] which is freely available at http://homer.ucsd.edu/homer/index.html. The whole genome was screened for LEF1 and TEAD motifs (LEF1(HMG)/H1-LEF1-ChIP-Seq(GSE64758)/Homer; TEAD(TEA)/Fibroblast-PU.1-ChIP-Seq(Unpublished)/Homer; TEAD1(TEAD)/HepG2-TEAD1-ChIP-Seq(Encode)/Homer; TEAD2(TEA)/Py2T-Tead2-ChIP-Seq(GSE55709)/Homer; TEAD3(TEA)/HepG2-TEAD3-ChIP-Seq(Encode)/Homer; TEAD4(TEA)/Tropoblast-Tead4-ChIP-Seq(GSE37350)/Homer) and the resulting genomic positions where filtered by co-appearance of both motifs around transcription start sites (TSS) with a maximum distance between the motifs of 100 bp. The filtered, annotated genomic positions were merged by *gene name* with the differential expression results of the RNA-Seq data. The resulting table contains genomic positions with predicted LEF1 and TEAD binding sites in the CIS regulatory elements of differentially expressed genes comparing SBcl2 melanoma cells with BRAFm-transduced NHEMs.

### Statistical analysis

Statistical analysis was performed using GraphPad Prism 10 software (GraphPad Software Inc., San Diego, CA, USA). If not otherwise stated, at least 3 biological replicates were measured. Statistical analysis was performed by multiple (Bonferroni-Dunn) or Student’s unpaired t-test. Comparison between more than two groups was determined using a one-way ANOVA (Bonferroni) or two-way ANOVA (Bonferroni or Tukey). All results are shown as mean ± SEM. A *p*-value < 0.05 was considered statistically significant.

## Results

### YAP activity in melanoma cells and during oncogene-induced senescence

**Fig. 1:**
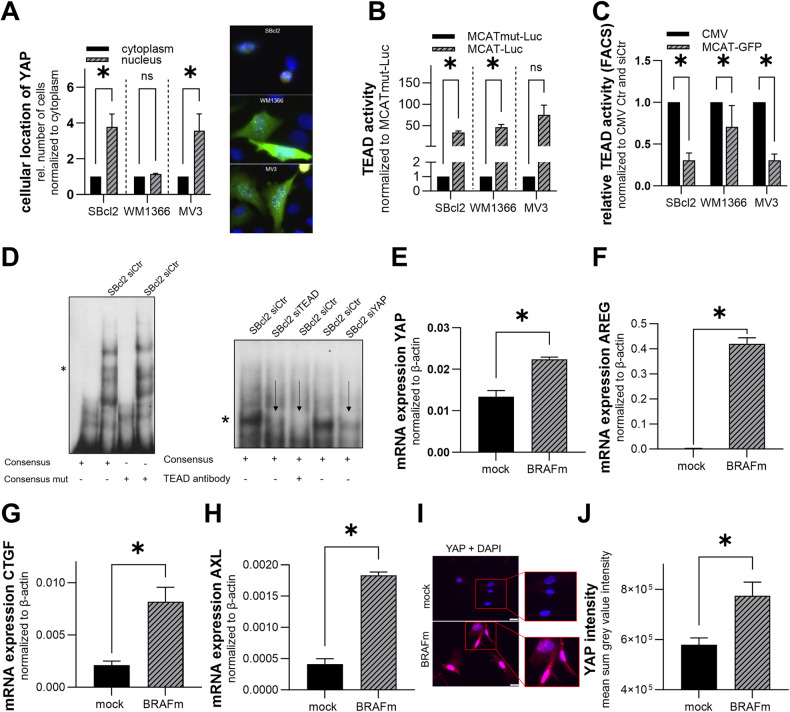
YAP activation in melanoma cells and during OIS. **A** Relative number of melanoma cells with nuclear or cytoplasmic YAP localization 48 h after the transfection of YAP reporter plasmid. Number of cells with cytoplasmic YAP accumulation was set as 1 (left). Values represent the mean ± SEM of 3 independent experiments (number of counted cells between 70 and 250 cells per treatment) (multiple *T*-tests (Bonferroni-Dunn) *P < 0.05, ns: not significant). Representative pictures of SBcl2, WM1366 and MV3 cells stained with DAPI (blue) and for YAP protein (green) (right). **B** Luciferase experiments with MCAT and MCATmut reporter constructs were performed with melanoma cell lines. MCATmut control was set as 1 for each cell line (*n* = 3). Bars are shown as mean ± SEM (multiple *T*-tests (Bonferroni-Dunn) *P < 0.05, ns: not significant). **C** Flow cytometry analysis with MCAT-eGFP and CMV control were performed with melanoma cell lines. CMV control was set as 100% for each cell line (*n* = 3). Bars are shown as mean ± SEM (multiple *T*-tests (Bonferroni-Dunn) *P < 0.05, ns: not significant). **D** EMSA with γ-^32^P-ATP-labeled MCAT consensus sequence (consensus) and mutated consensus sequence (consensus mut) oligonucleotide containing one predicted TEAD binding site. SBcl2 cells were treated with siTEAD (lane 2), siYAP (lane 5) or anti-TEAD antibody (lane 3) prior to loading. Star: TEAD-oligo binding. Arrow: reduction of the TEAD oligo band. **E** Expression of YAP mRNA in mock/BRAFm-transduced NHEMs cells normalized to β-actin measured by qRT-PCR (*n* = 3). Bars are shown as mean ± SEM (students *T*-test *P < 0.05, ns: not significant). **F**–**H** mRNA expression of classical YAP target genes AREG, AXL and CTGF in mock/BRAFm-transduced NHEMs normalized to β-actin measured by qRT-PCR (*n* = 3). Bars are shown as mean ± SEM (students *T*-test *P < 0.05, ns: not significant). **I** Representative immunofluorescence images of mock/BRAFm-transduced NHEMs stained with DAPI (blue) and an anti-YAP antibody (red). **J** Nuclear YAP intensity in mock/BRAFm-transduced NHEMs (*n* = 3). Bars are shown as mean ± SEM (students *T*-test *P < 0.05, ns: not significant).

Based on studies showing that the mechanosensitive YAP effector system activates the expression of pro-proliferative and survival-enhancing genes and therefore plays an important role in tumor progression and cancer development [48–50], we analyzed YAP localization and activity in melanoma. As the active YAP protein translocates into the nucleus, we first investigated the localization of the YAP protein in melanoma cells. In SBcl2 and MV3 cell lines, we observed a significantly greater percentage of cells with nuclear YAP than cells with cytoplasmic YAP protein (Fig. 1A). In WM1366 cells, the percentage of cells that contained nuclear or cytoplasmic YAP protein was similar. To validate the transcriptional activity of YAP, we performed MCAT luciferase reporter assays and MCAT-eGFP flow cytometry analyses in different melanoma cell lines. All three analyzed melanoma cell lines showed strong general TEAD reporter activity (Fig. 1B). Interestingly, using flow cytometry analysis, we revealed that in ~30% (SBcl2 and MV3) and 60% (WM1366) of the cells, YAP was active (Fig. 1C).

Next, we transfected SBcl2 cells with a pool of siRNAs against TEAD or YAP. Transfection of melanoma cell lines led to a reduction in TEAD or YAP mRNA expression of ~80–90% after 48 h (Supplemental Fig. S1A/B). Additionally, at the protein level, siTEAD and siYAP reduced protein expression by ~70–80% after 48 h (Supplemental Fig. S1C-F; uncropped blots and molecular weight markers are shown in Fig. S4). To further analyze these siRNA pool-treated cells, we utilized EMSA and confirmed the direct interaction between YAP-TEAD and the DNA (Fig. 1D). Moreover, by using an oligonucleotide containing TEAD binding motifs, knockdown of TEAD and YAP led to a loss in DNA interactions, as measured by a decrease in the intensity of the YAP-TEAD-oligo band compared to that in control cells. An anti-TEAD antibody that competes with DNA-binding also led to displacement of the YAP-TEAD-oligo band. Thus, we clearly demonstrated the activation of YAP signaling and the interaction of YAP with TEAD in melanoma cells.

To determine the impact of YAP activity on melanoma development, we analyzed the role of YAP in normal melanocytes. Based on studies showing that the YAP/TAZ system can modulate senescence induction in hepatocytes and fibroblasts [51], we focused on melanocytic oncogene-induced senescence (OIS). To mimic the status of OIS in normal human epidermal melanocytes (NHEMs), lentiviral transduction of mutated BRAF^V600E^ (BRAFm) was used to induce OIS as initially described by Michaloglou et al. (10). Via qRT‒PCR analyses, we observed significantly greater YAP mRNA expression in BRAFm-transduced melanocytes than in mock-transduced control cells after 7 days (Fig. 1E). We further revealed that the expression of classical YAP target molecules, such as AREG, AXL and CTGF, was significantly induced in OIS melanocytes (Fig. 1F-H). Immunofluorescence staining of the YAP protein showed strong nuclear accumulation during BRAFm-induced senescence in melanocytes (Fig. 1I). Statistical analysis confirmed the nuclear accumulation of YAP in senescent melanocytes (Fig. 1J).

Taken together, these results illustrate that YAP signaling is already activated during OIS in melanocytes and is additionally activated in melanoma.

### Wnts and their role in OIS and melanoma

**Fig. 2:**
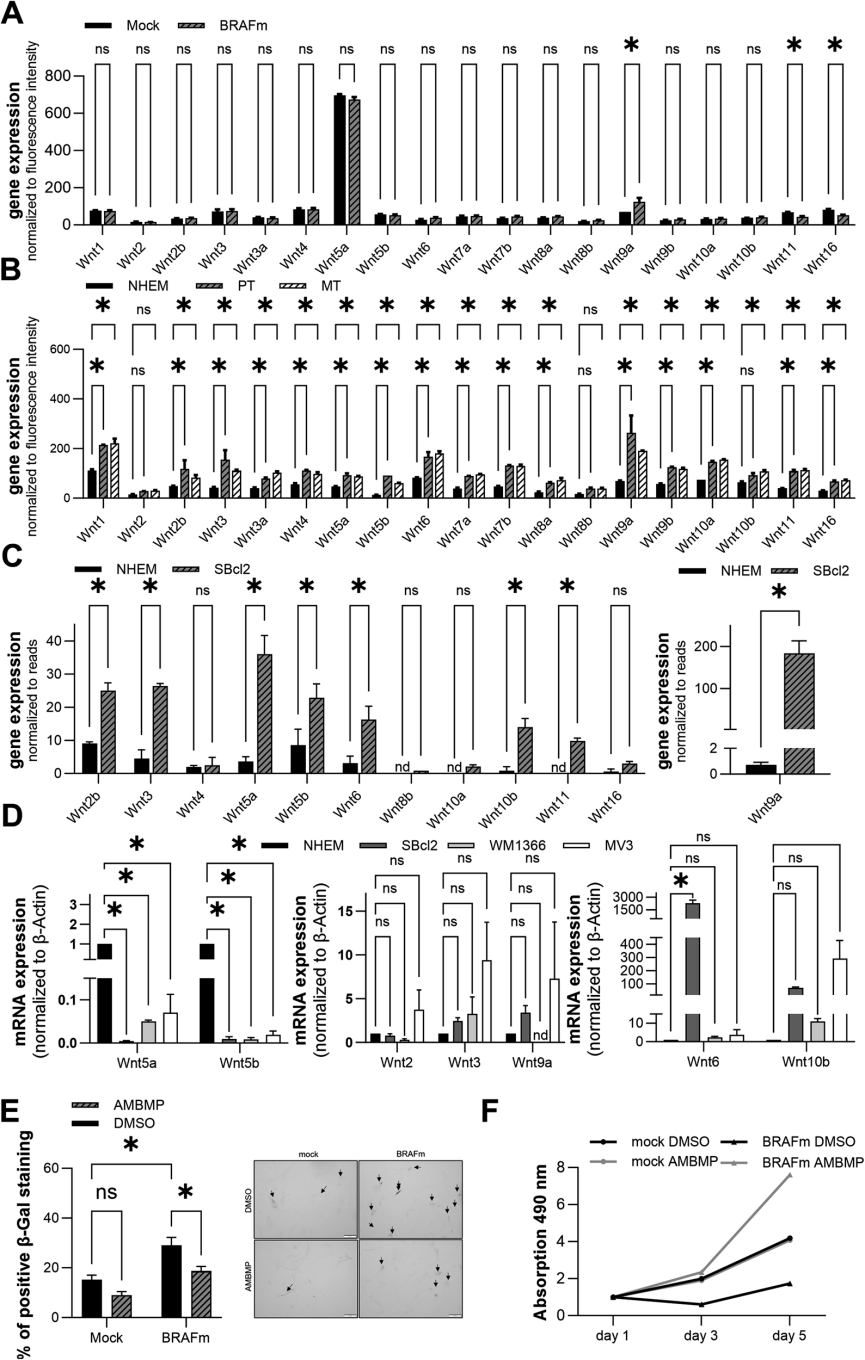
Wnt expression in mock/BRAFm-transduced NHEMs and melanoma cells. **A** Wnt expression level in mock/BRAFm-transduced NHEMs analyzed by cDNA array (*n* = 3). Bars are shown as mean ± SEM (two-way ANOVA (Bonferroni) *P < 0.05, ns: not significant). **B** Wnt expression level in primary and metastatic melanoma cells compared to NHEMs analyzed by cDNA array (GEO: GSE108969) (*n* = 2). Bars are shown as mean ± SEM (two-way ANOVA (Bonferroni) *P < 0.05, ns: not significant). **C** Wnt RNA-Seq read counts normalized to library size in SBcl2 cells compared to NHEMs. **D** Relative Wnt mRNA expression in NHEMs and SBcl2, WM1366 and MV3 cells measured by qRT-PCR. mRNA level in NHEMs is set to 1 (*n* = 2). Bars are shown as mean ± SEM (two-way ANOVA (Bonferroni) *P < 0.05, ns: not significant). **E** Percentages of SA-β-Galactosidase positive cells in mock/BRAFm-transduced NHEMs after the treatment with AMBMP agonist or DMSO control (left) (*n* = 3). Bars are shown as mean ± SEM (two-way ANOVA (Tukey) *P < 0.05, ns: not significant). Example image of light microscopic examination of SA-β-Galactosidase staining (right). **F** Representative proliferation curves of mock/BRAFm-transduced NHEMs after treatment with AMBMP or DMSO control resulting from a XTT assay.

Wnts are known to be important in melanoma progression [19]. We investigated whether aberrant expression of Wnts can be found in NHEMs compared to BRAFm-transduced melanocytes or in early-stage melanoma to determine whether changes in Wnt expression are involved in melanoma development. Analyses of cDNA arrays comparing mRNA expression in mock and BRAFm cells revealed no significant changes in the expression of most Wnt mRNAs, except for Wnt9a, Wnt11 and Wnt16 (Fig. 2A). In contrast, analyses of a melanoma cDNA array (GEO: GSE108969) showed that the mRNA levels of nearly all Wnts (except Wnt2 and Wnt8b) were significantly greater than those in NHEMs and primary tumors and metastases (Fig. 2B) [52]. Supporting these data, RNA sequencing (RNA-Seq) analysis revealed a strong increase in Wnt RNA-Seq reads in SBcl2 melanoma cells compared to those in NHEMs (Fig. 2C) (27). Furthermore, we confirmed these data in the melanoma cell lines SBcl2, WM1366, and MV3. Therefore, we used quantitative RT‒PCR to determine the mRNA levels of selected Wnts (Fig. 2D). As expected, we observed strong induction of some Wnts, whereas others, such as Wnt5a and Wnt5b, which were upregulated in several melanoma cell lines, were surprisingly downregulated in the analyzed melanoma cell lines compared to those in NHEMs.

Next, we wanted to investigate whether the general upregulation of Wnt signaling influences OIS. Therefore, we treated BRAFm-transduced NHEMs with the Wnt agonist AMBMP to induce β-catenin- and TCF-dependent transcriptional activity [53]. The activity of senescence-associated β-Galactosidase (SA-β-Gal) is the gold standard for analyzing cellular senescence [39]. We used this method to quantify senescent melanoma cells after treatment with AMBMP compared to the control cells. SA-β-Gal staining revealed a significant decrease in the number of β-Gal-positive cells caused by treatment with AMBMP in BRAFm-transduced NHEMs compared to that in the DMSO control (Fig. 2E). We observed no changes in the mock-transduced NHEMs after AMBMP treatment. An XTT assay revealed greater cell viability in BRAFm-transduced NHEMs treated with AMBMP than in those treated with DMSO, indicating the induced proliferation of these senescent cells (Fig. 2F). In summary, the activation of Wnt signaling in BRAFm-transduced NHEMs reduced the incidence of senescence in BRAFm melanocytes.

### Role of Wnt6 in OIS and melanoma

As we were able to show that Wnts play a role in breaking OIS, we focused on Wnt6, as it was upregulated the most in the tested melanoma cells compared to the NHEMs in OIS.

**Fig. 3:**
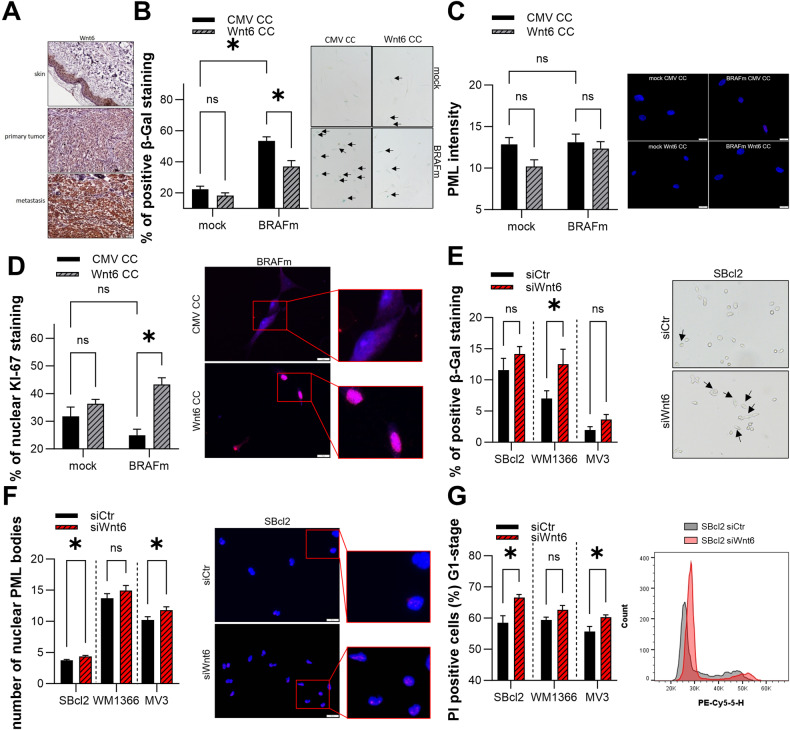
Effect of Wnt6 on OIS and its role in melanoma cells. **A** Representative immunohistochemical staining of Wnt6 protein in normal human skin, primary human melanoma and human metastatic melanoma tissue samples (*n* = 10). **B** Percentages of SA-β-Galactosidase positive cells (blue) in mock/BRAFm-transduced NHEMs co-cultured with Wnt6 OE HEK (Wnt6 CC) or with CMV OE HEK (CMV CC) cells (left) (*n* = 3). Bars are shown as mean ± SEM (two-way ANOVA (Tukey) *P < 0.05, ns: not significant). Exemplary image of light microscopic examination of SA-β-Galactosidase staining (right). **C** Immunofluorescence staining of PML and DAPI in mock/BRAFm-transduced NHEMs co-cultured with Wnt6 OE HEK (Wnt6 CC) or with CMV OE HEK (CMV CC) cells (*n* = 3). Bars are shown as mean ± SEM (two-way ANOVA (Tukey) *P < 0.05, ns: not significant). **D** Immunofluorescence staining of KI-67 and DAPI in mock/BRAFm-transduced NHEMs co-cultured with Wnt6 OE HEK (Wnt6 CC) or with CMV OE HEK (CMV CC) cells. The graph shows the nuclear accumulation of KI-67 (left) (*n* = 3). Bars are shown as mean ± SEM (two-way ANOVA (Tukey) *P < 0.05, ns: not significant). Sample image of overlays of KI-67 (purple) and DAPI (blue) (right). **E** Percentages of SA-β-Galactosidase positive cells (blue) in SBcl2, WM1366 and MV3 cell lines 48 h after transfection with siWnt6 or siCtr (left) (*n* = 3). Bars are shown as mean ± SEM (multiple *T*-tests (Bonferroni-Dunn)*P < 0.05, ns: not significant). Example image of light microscopic examination of SA-β-Galactosidase staining in SBcl2 cells (right). **F** Immunofluorescence staining of PML and DAPI in melanoma cell lines 48 h after the transfection with siWnt6 or siCtr. The graph shows the number of nuclear PML bodies (left) (*n* = 3). Bars are shown as mean ± SEM (multiple *T*-tests (Bonferroni-Dunn) *P < 0.05, ns: not significant). Example image of overlays of PML (red) and DAPI (blue) in SBcl2 (right). **G** Cell cycle analysis 48 h after Wnt6 inhibition in SBcl2, WM1366 and MV3 cells. Bars represent cells in G1 phase (left) (*n* = 3). Bars are shown as mean ± SEM (multiple *T*-tests (Bonferroni-Dunn) *P < 0.05, ns: not significant). Representative histograms of cell cycle analysis in SBcl2 cells 48 h after siWnt6 or siCtr transfection.

Immunohistochemical staining of Wnt6 in human skin, primary tumor tissue and metastatic tissue revealed strong staining (Fig. 3A), which therefore corresponded to our results on the mRNA expression levels detected via qRT‒PCR. In co-culture experiments, we tested the effect of Wnt6 overexpression in melanocytes by transfecting HEK293T cells with OIS. Therefore, we cultured BRAFm- or mock-transduced NHEMs from day four after transduction together with HEK293T cells, which were transfected with an active Wnt6 overexpression plasmid (Wnt6 OE HEK) or a control plasmid (CMV OE HEK), respectively. SA-β-Gal staining revealed a reduction in the number of β-Gal-positive cells among BRAFm-transduced NHEMs due to co-culture with Wnt6 OE HEK cells compared to co-culture with CMV OE HEK cells (Fig. 3B). To further analyze the influence of Wnt6 OE on OIS, we revealed decreased levels of nuclear promyelocytic leukemia protein (PML) via immunofluorescence staining, a molecular marker of slow-growing and less dividing cells. Compared with co-culture with CMV OE HEK, PML immunofluorescence staining of co-culture mock/BRAFm-transduced NHEMs with Wnt6 OE HEK tended to reveal a decrease in nuclear PML intensity (Fig. 3C). Immunofluorescence staining for KI-67, a marker of proliferation, revealed a significantly greater percentage of cells stained for nuclear KI-67 in co-cultured mock/BRAFm-transduced NHEMs with Wnt6 OE HEK than in BRAFm-transduced NHEMs with CMV OE HEK (Fig. 3D).

Based on the effects of Wnt6 on OIS in melanocytes, we investigated the function of Wnt6 in melanoma cells and determined the status of senescence after the knockdown of Wnt6. To confirm a mutation-independent mechanism, melanoma cell lines without BRAF mutation were used. Key experiments were also carried out on BRAFmutant melanoma cell lines and are shown in the supplement. Transfection of melanoma cell lines with a pool of siRNAs against Wnt6 led to a reduction in Wnt6 mRNA expression of ~40% after 48 h (Supplemental Fig. S1G). SA-β-Gal staining revealed an increased number of β-Gal-positive cells after knockdown of Wnt6 compared to that in control cells for the SBcl2, WM1366 and MV3 cell lines (Fig. 3E, Supplemental Fig. S2A). PML protein expression in the cell lines tended to increase the relative number of nuclear PML bodies in siWnt6-treated cells compared to that in siCtr-treated cells in WM1366 cells, and there was a significant increase in the expression of SBcl2 and MV3 (Fig. 3F, Supplemental Fig. S2B). Another phenotypical marker for cellular senescence is the induction of G1 arrest according to cell cycle analysis. Therefore, we performed flow cytometric analysis of the cell cycle to identify potential G1 arrest after the knockdown of Wnt6. The results showed that, compared with siCtr, siWnt6 significantly induced G1 arrest in the SBcl2 and MV3 cell lines (Fig. 3G). In the WM1366 cell line, a trend toward G1 arrest was detected.

### Role of Wnt10b in OIS and melanoma

**Fig. 4:**
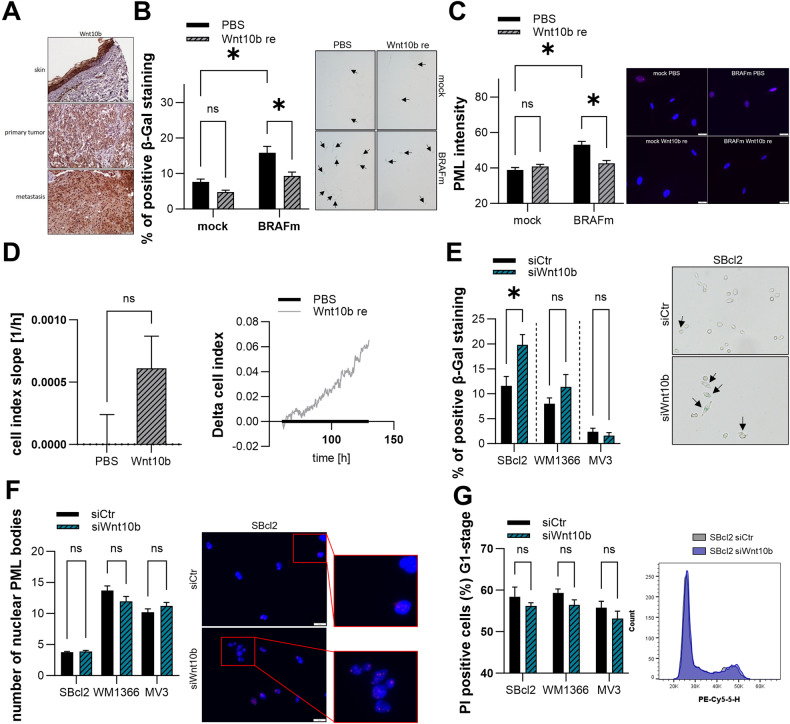
Role of Wnt10b on OIS and in melanoma cells. **A** Representative immunohistochemical staining of Wnt10b protein in normal human skin, primary human melanoma, and human metastatic melanoma tissue samples (*n* = 10). **B** Percentages of SA-β-Galactosidase positive cells (blue) in mock/BRAFm-transduced NHEMs treated with recombinant Wnt10b or PBS (left) (*n* = 3). Bars are shown as mean ± SEM (two-way ANOVA (Tukey) *P < 0.05, ns: not significant). Example image of light microscopic examination of SA-β-Galactosidase staining (right). **C** Immunofluorescence staining’s of PML and DAPI in mock/BRAFm-transduced NHEMs treated with recombinant Wnt10b or PBS. The graph shows nuclear accumulation of PML (left) (*n* = 3). Bars are shown as mean ± SEM (two-way ANOVA (Tukey) *P < 0.05, ns: not significant). Sample image of overlays of PML (red) and DAPI (blue) (right). **D** Representative real-time cell proliferation curves of BRAFm-transduced NHEMs treated with recombinant Wnt10b or PBS (BRAFm PBS set as 1) (right panel) and quantified “slope” (proliferative ability) (left panel) (*n* = 3). Bars are shown as mean ± SEM (multiple *T*-tests (Bonferroni-Dunn) *P < 0.05, ns: not significant). **E** Percentages of SA-β-Galactosidase positive cells (blue) in SBcl2, WM1366, and MV3 cell lines 48 h after transfection with siWnt10b or siCtr (left) (*n* = 3). Bars are shown as mean ± SEM (multiple *T*-tests (Bonferroni-Dunn) *P < 0.05, ns: not significant). Example image of light microscopic examination of SA-β-Galactosidase staining in SBcl2 cells (right). **F** Immunofluorescence staining of PML and DAPI in melanoma cell lines 48 h after the transfection with siWnt10b or siCtr. The graph shows the number of nuclear PML bodies (left) (*n* = 3). Bars are shown as mean ± SEM (multiple *T*-tests (Bonferroni-Dunn) *P < 0.05, ns: not significant). Sample image of overlays of PML (red) and DAPI (blue) in SBcl2 cells (right). **G** Cell cycle analysis 48 h after Wnt10b inhibition in SBcl2, WM1366, and MV3 cells. Bars represent cells in G1 phase (left) (*n* = 3). Bars are shown as mean ± SEM (multiple *T*-tests (Bonferroni-Dunn) *P < 0.05, ns: not significant). Representative histograms of cell cycle analysis in SBcl2 cells 48 h after siWnt10b or siCtr transfection.

Although we previously showed that Wnt6 reduces OIS in melanocytes but induces senescence in melanoma cell lines, we wanted to determine whether Wnt10b, which is also upregulated in melanoma cell lines, has a similar function. Immunohistochemical staining of Wnt10b in human skin, primary tumor tissue and metastatic tissue revealed increased staining in melanoma tissue compared to skin (Fig. 4A), which was consistent with our qRT‒PCR results on the mRNA expression levels. First, we investigated the role of Wnt10b in a melanocyte model of OIS. Here, we treated mock/BRAFm NHEMs with recombinant Wnt10b and further analyzed the cells via different senescence-associated assays. SA-β-Gal staining revealed a significantly reduced number of β-Gal-positive cells after treatment with recombinant Wnt10b in BRAFm-transduced NHEMs compared to that in the PBS control (Fig. 4B). Treatment of mock NHEMs with recombinant Wnt10b tended to reduce the number of β-Gal-positive cells compared to that in the PBS control group. We also confirmed the effect of recombinant Wnt10b on senescence via PML immunofluorescence staining (Fig. 4C). The results showed a significant reduction in the PML intensity in the recombinant Wnt10b treatment group compared to the PBS control group. In addition, treatment with recombinant Wnt10b resulted in increased cell growth in BRAFm-transduced NHEMs compared to control cells, as measured by RTCA (Fig. 4D). Taken together, these results confirm that Wnt10b leads to a disruption of OIS in melanocytes, similar to the previously described effects of Wnt6. Based on the effect of Wnt10b on OIS in melanocytes, we then investigated the function of Wnt10b in melanoma cell lines and determined the status of senescence after the knockdown of Wnt10b. Transfection of melanoma cell lines with a pool of siRNAs against Wnt10b led to a reduction in Wnt10b mRNA expression of ~40% after 48 h (Supplemental Fig. S1H). SA-β-Gal staining revealed strong induction of β-Gal-positive cells after the knockdown of Wnt10b in comparison to that in control cells in the SBcl2 and WM1366 cell lines (Fig. 4E, Supplemental Fig. S2C). Compared with siCtr, knockdown of Wnt10b did not influence the number of β-Gal-positive cells in the MV3 population. These effects could not be reproduced with PML immunostaining (Fig. 4F, Supplemental Fig. S2D) or cell cycle analysis (Fig. 4G). In both experiments, knockdown of Wnt10b had no effect on senescence in melanoma cell lines compared to that in the control cells.

### Alternative YAP-β-catenin signaling axis

**Fig. 5:**
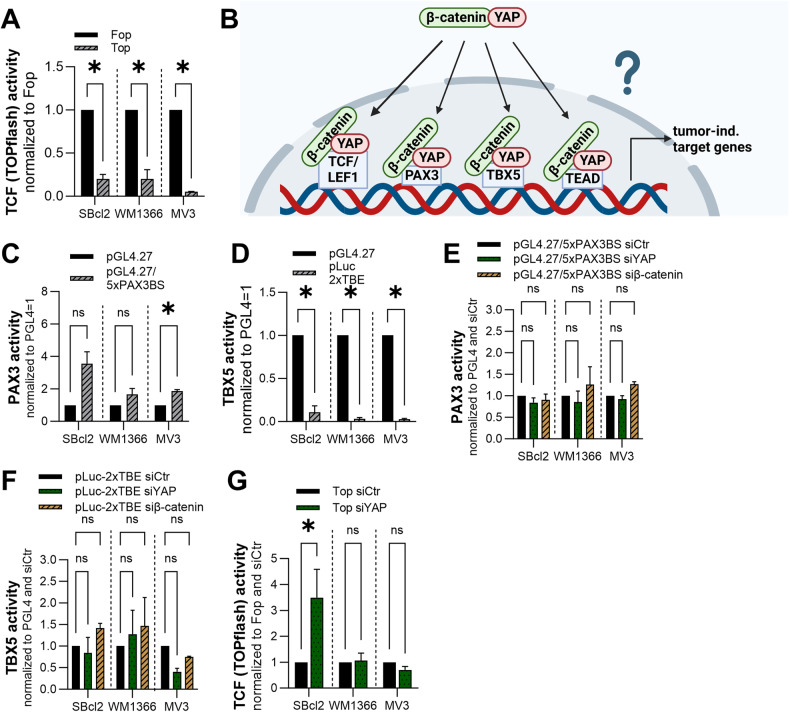
Transcriptional activity of TCF/LEF1, PAX3 and TBX5. **A** Luciferase experiments with TOPflash and FOPflash reporter constructs were performed with melanoma cell lines. FOPflash control transfection was set as 1 for each cell line (*n* = 3). Bars are shown as mean ± SEM (multiple *T*-tests (Bonferroni-Dunn) *P < 0.05, ns: not significant). **B** Schematic illustration of the hypothesis (created with BioRender). **C** Luciferase experiments with PAX3 and PGL4.27 reporter constructs were performed with SBcl2, WM1366 and MV3 cells. PGL4.27 control transfection was set as 1 for each cell line (*n* = 3). Bars are shown as mean ± SEM (multiple *T*-tests (Bonferroni-Dunn) *P < 0.05, ns: not significant). **D** Luciferase experiments with TBE and PGL4.27 reporter constructs were performed with SBcl2, WM1366 and MV3 cells. PGL4.27 control transfection was set as 1 for each cell line (*n* = 3). Bars are shown as mean ± SEM (multiple *T*-tests (Bonferroni-Dunn) *P < 0.05, ns: not significant). **E** Luciferase experiments with PAX3 and PGL4.27 reporter constructs were performed with SBcl2, WM1366 and MV3 cells 48 h after transfection of siYAP, siβ-catenin or siCtr. Values were normalized to PGL4.27 control transfection and siCtr was set as 1 for each cell line (*n* = 3). Bars are shown as mean ± SEM (multiple *T*-tests (Bonferroni-Dunn) *P < 0.05, ns: not significant). **F** Luciferase experiments with TBE and PGL4.27 reporter constructs were performed with SBcl2, WM1366 and MV3 cells 48 h after transfection of siYAP, siβ-catenin or siCtr. Values were normalized to PGL4.27 control transfection and siCtr was set as 1 for each cell line (*n* = 3). Bars are shown as mean ± SEM (multiple *T*-tests (Bonferroni-Dunn) *P < 0.05, ns: not significant). **G** Luciferase experiments with TOPflash and FOPflash reporter constructs were performed with melanoma cell lines 48 h after siYAP or siCtr transfection. Values were normalized to FOPflash control transfection and siCtr was set as 1 for each cell line (*n* = 3). Bars are shown as mean ± SEM (multiple *T*-tests (Bonferroni-Dunn) *P < 0.05, ns: not significant).

The major downstream pathway of Wnts is dependent on β-catenin. To further understand the molecular mechanism by which OIS is disrupted by activating Wnt signaling during early melanoma development, we investigated the downstream effector β-catenin and evaluated its activity via luciferase reporter assays. Interestingly, compared with the control cells, all the analyzed cell lines did not exhibit classical β-catenin reporter activity (Fig. 5A). These data confirmed our previously published findings on β-catenin in additional melanoma cell lines [54].

As we clearly revealed no β-catenin/LEF1/TCF activity in several melanoma cell lines, a literature search suggested that β-catenin also acts as a co-factor for YAP signaling [55]. Therefore, we analyzed whether β-catenin and YAP form complexes in melanoma cells and whether these complexes can interact with different transcription factors, such as paired box gene 3 (PAX3), T-box transcription Factor 5 (TBX5), TCF/LEF1 or TEAD, causing a switch from senescence-stabilized target genes to tumor-supportive target genes (Fig. 5B). First, we investigated the transcriptional activity of PAX3 or TBX5 in the melanoma cell lines SBcl2, WM1366 and MV3 using reporter gene assays. These results showed only slight activity of PAX3 (Fig. 5C) and no activity of TBX5 (Fig. 5D) compared to those of the empty pGL4.27 construct. Next, we transfected melanoma cell lines with an siRNA pool against YAP or β-catenin. Transfection of melanoma cell lines led to a reduction in β-catenin mRNA expression of ~80–90% and protein expression of ~70–80% after 48 h (Supplemental Fig. S1I-K; uncropped blots and molecular weight markers are shown in Fig. S5). Neither the knockdown of YAP nor the knockdown of β-catenin modified PAX3 (Fig. 5E) or TBX5 reporter activity (Fig. 5F) compared to that in control cells. Furthermore, the results of the luciferase reporter assay showed that, compared with the control treatment, the knockdown of YAP did not induce classical β-catenin activity, except in SBcl2 cells (Fig. 5G). Thus, we assumed that neither PAX3 nor TBX5 transcriptional activity plays a major role in the β-catenin- or YAP-induced effects.

### Interaction between YAP and β-catenin

**Fig. 6:**
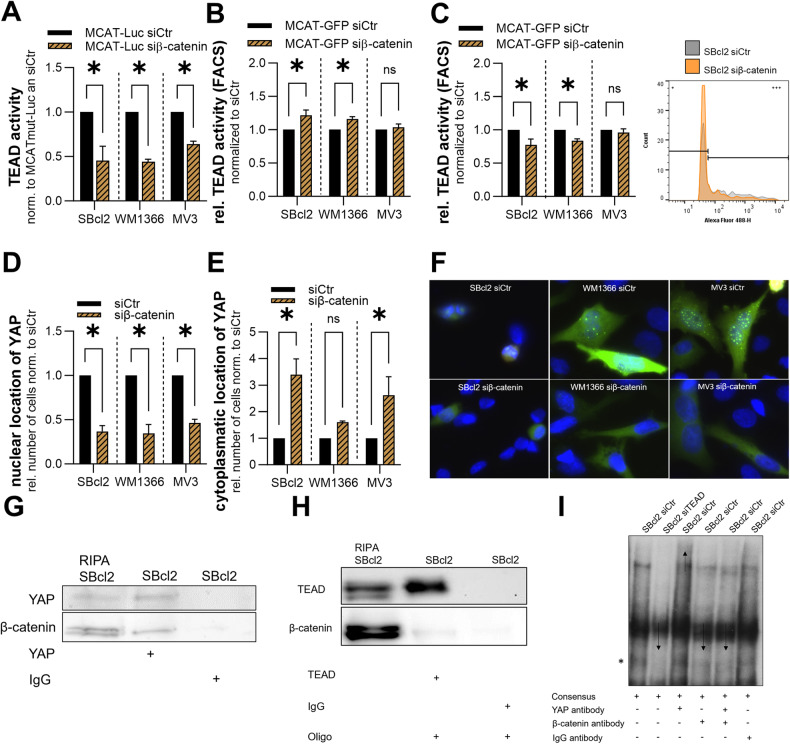
Alternative YAP-β-catenin signaling axis. **A** Luciferase experiments with MCAT and MCATmut reporter constructs were performed with melanoma cell lines 48 h after siβ-catenin or siCtr transfection. Values were normalized to MCATmut control transfection and siCtr was set as 1 for each cell line (*n* = 3). Bars are shown as mean ± SEM (multiple *T*-tests (Bonferroni-Dunn) *P < 0.05, ns: not significant). **B**, **C** Flow cytometry analyses with MCAT-eGFP were performed with melanoma cell lines 48 h after siβ-catenin or siCtr transfection. **B** Analyses of high GFP-staining in melanoma cells. siCtr was set as 1 for each cell line. **C** Analyses of low GFP-staining in melanoma cells. siCtr was set as 1 for each cell line (left) (*n* = 3). Bars are shown as mean ± SEM (multiple *T*-tests (Bonferroni-Dunn) *P < 0.05, ns: not significant). Representative histograms of GFP-staining’s in SBcl2 cells 48 h after siβ-catenin or siCtr transfection. Cells were gated into low (+) and high GFP-staining (+++). **D** Relative number of cells with nuclear YAP localization 48 h after transfection of siβ-catenin or siCtr visualized with YAP localization plasmid. siCtr was set as 1 (*n* = 3). Bars are shown as mean ± SEM (multiple *T*-tests (Bonferroni-Dunn) *P < 0.05, ns: not significant). **E** Relative number of cells with cytoplasmic YAP localization 48 h after transfection of siβ-catenin or siCtr visualized with YAP localization plasmid. siCtr was set as 1 (*n* = 3). Bars are shown as mean ± SEM (multiple *T*-tests (Bonferroni-Dunn) *P < 0.05, ns: not significant). **F** Representative pictures of SBcl2, WM1366 and MV3 cells stained with DAPI (blue) and YAP (green) (right). **G** Interaction of YAP and β-catenin measured by co-immunoprecipitation with anti-YAP antibody in SBcl2 cells (lane 2). Lane 1 represents input of SBcl2 cells and lane 3 immunoprecipitation with anti-IgG antibody in SBcl2 cells. Immunoblotting with anti-YAP and anti β-catenin antibodies. **H** Interaction of TEAD, YAP and β-catenin analyzed by co-immunoprecipitation with anti-TEAD antibody in SBcl2 cells (lane 2). Lane 1 represents input of SBcl2 cells and lane 3 immunoprecipitation with anti-IgG antibody in SBcl2 cells. Immunoblotting with anti-TEAD and anti β-catenin antibodies. **I** EMSA with γ-^32^P-ATP-labeled MCAT consensus sequence (consensus) oligonucleotide containing one predicted TEAD binding sites. Analysis of TEAD-Oligo band was performed with siTEAD (lane 2), anti-YAP antibody (lane 3, 5) and anti-β-catenin antibody (lane 4, 5). Star: TEAD-oligo binding. Arrow: reduction of the TEAD-Oligo band or supershift due to complexing of YAP-β-catenin-TEAD.

The transcription factor family TEAD1-4 is the commonly known transcription factor family that forms DNA-binding complexes with YAP and is therefore another potential interaction partner of β-catenin and YAP. In further experiments, we focused on TEAD and its interaction with YAP and β-catenin. We transfected the melanoma cell lines SBcl2, WM1366 and MV3 with an siRNA pool against β-catenin and then performed MCAT luciferase reporter assays to measure TEAD activity. The results revealed a significant reduction in YAP/TEAD transcriptional activity in all cell lines after siβ-catenin transfection compared to siCtr transfection (Fig. 6A). To validate these results, we measured YAP/TEAD activity by flow cytometry analysis. Compared to siCtr, treatment with a pool of siRNAs against β-catenin caused a significant reduction in highly YAP/TEAD-controlled GFP expression in the SBcl2 and WM1366 cells (Fig. 6B). Supporting our results, we detected a significant increase in the number of cells with a decreased fluorescence signal after the knockdown of β-catenin compared to that in the siCtr group in these two cell lines (Fig. 6C). There was no difference in YAP activity in MV3 cells due to the knockdown of β-catenin compared to that in siCtr cells according to flow cytometry analyses. However, the results in SBcl2- and WM1366 cells led us to hypothesize that YAP and β-catenin form a complex and that the knockdown of β-catenin reduces YAP activity.

Next, we were interested in the localization of the YAP protein after treatment with siβ-catenin and observed a shift in the level of the YAP protein from the nucleus to the cytoplasm caused by the knockdown of β-catenin (Fig. 6D-F). Similarly, a significantly reduced relative number of cells with nuclear YAP was detected in the SBcl2, WM1366 and MV3 cells transfected with siβ-catenin (Fig. 6D). Analysis of all the cell lines showed that the amount of the YAP protein in the cytoplasm was greater in the siβ-catenin-transfected cells than in the siCtr cells (Fig. 6E).

Since we are able to modulate YAP activity by β-catenin knockdown, we investigated whether there is a direct interaction between YAP and β-catenin in melanoma cells. Co-immunoprecipitation experiments with an anti-YAP antibody detected an endogenous YAP-β-catenin complex in SBcl2 cells as well as in WM1366 and MV3 cells (Fig. 6G and Supplemental Fig. S3A–D). In further experiments, we detected β-catenin after co-immunoprecipitation of SBcl2 cells with an anti-TEAD antibody (Fig. 6H). Consequently, we were able to confirm the presence of the TEAD-YAP-β-catenin complex in melanoma cells.

To evaluate the localization of this complex, we performed EMSA using a TEAD-binding motif (Fig. 6I). In nuclear extracts of cells after siTEAD transfection, we detected no binding of the ternary complex to the DNA oligonucleotide, revealing that TEAD is essential for a potential DNA interaction involving the TEAD-YAP-β-catenin complex (Fig. 6I Lane 2). A YAP antibody led to a supershift, confirming the interaction of YAP-TEAD with DNA (Fig. 6I, Lane 5). The addition of a β-catenin antibody led to a reduction in the intensity of the YAP-TEAD-oligo band (Fig. 6I Lane 4). Similarly, the addition of both antibodies (against YAP and β-catenin) led to displacement of the YAPTEAD-oligo band (Fig. 6I Lane 5). In summary, we demonstrated that TEAD-YAP-β-catenin interacts with DNA and that knockdown of β-catenin results in a reduction in YAP activity via TEAD.

### Knockdown of YAP and β-catenin and its influence on senescence in melanoma cells

**Fig. 7:**
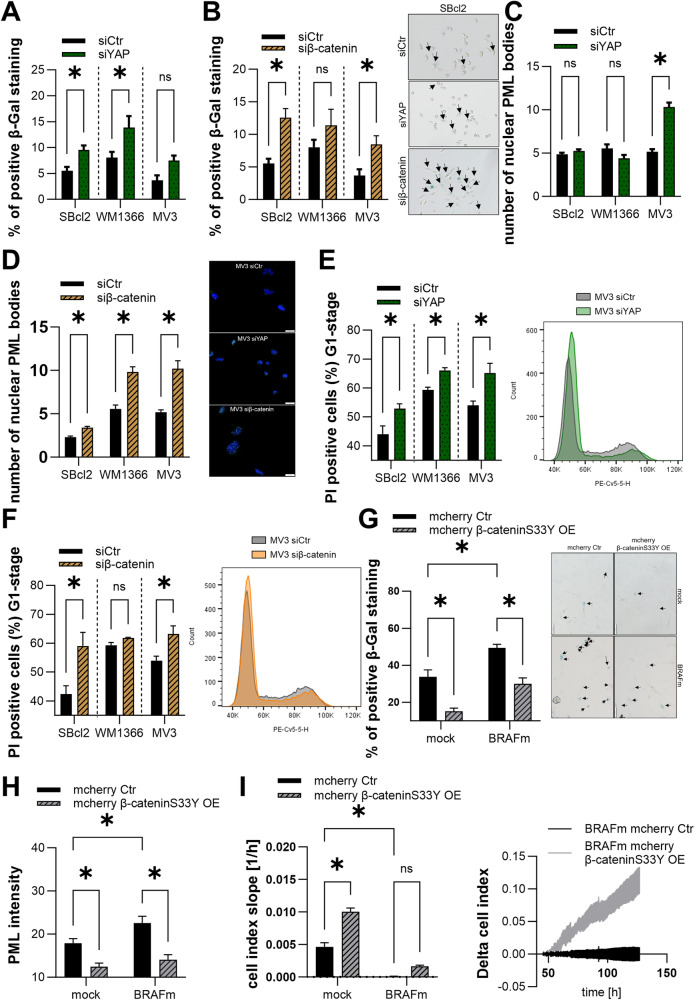
Influence of YAP and β-catenin on OIS. **A** Percentages of SA-β-Galactosidase positive cells (blue) in SBcl2, WM1366, and MV3 cell lines 48 h after transfection with siYAP or siCtr (left) (n = 3). Bars are shown as mean ± SEM (multiple *T*-tests (Bonferroni-Dunn) *P < 0.05, ns: not significant). Sample image of light microscopic examination of SA-β-Galactosidase staining in SBcl2 cells (right). **B** Percentages of SA-β-Galactosidase positive cells (blue) in SBcl2, WM1366, and MV3 cell lines 48 h after transfection with siβ-catenin or siCtr (left) (*n* = 3). Bars are shown as mean ± SEM (multiple *T*-tests (Bonferroni-Dunn) *P < 0.05, ns: not significant). Example image of light microscopic examination of SA-β-Galactosidase staining in SBcl2 cells (right). **C**Immunofluorescence staining of PML and DAPI in melanoma cell lines 48 h after the transfection with siYAP or siCtr. The graph shows the number of nuclear PML bodies (left). Representative image of overlays of PML (red) and DAPI (blue) in MV3 cells (right). **D** Immunofluorescence staining of PML and DAPI in melanoma cell lines 48 h after the transfection with siβ-catenin or siCtr. The graph shows the number of nuclear PML bodies (left) (*n* = 3). Bars are shown as mean ± SEM (multiple *T*-tests (Bonferroni-Dunn) *P < 0.05, ns: not significant). Example image of overlays of PML (red) and DAPI (blue) in MV3 cells (right). **E** Cell cycle analysis 48 h after YAP inhibition in SBcl2, WM1366 and MV3 cells. Bars represent cells in G1 phase (left) (*n* = 3). Bars are shown as mean ± SEM (multiple *T*-tests (Bonferroni-Dunn) *P < 0.05, ns: not significant). Representative histograms of cell cycle analysis in MV3 cells 48 h after siYAP or siCtr transfection. **F** Cell cycle analysis 48 h after β-catenin inhibition in SBcl2, WM1366 and MV3 cells. Bars represent cells in G1 phase (left) (*n* = 3). Bars are shown as mean ± SEM (multiple *T*-tests (Bonferroni-Dunn) *P < 0.05, ns: not significant). Representative histograms of cell cycle analysis in MV3 cells 48 h after siβ-catenin or siCtr transfection. **G** Percentages of SA-β-Galactosidase positive cells (blue) in mock/BRAFm-, mcherry/mcherry β-cateninS33Y OE double-transduced NHEMs (left) (*n* = 3). Bars are shown as mean ± SEM (two-way ANOVA (Tukey) *P < 0.05, ns: not significant). Sample image of light microscopic examination of SA-β-Galactosidase staining (right). **H** Immunofluorescence stainings of PML and DAPI in mock/BRAFm-transduced, mcherry/mcherry β-cateninS33Y OE double transduced NHEMs. The graph shows nuclear accumulation of PML (*n* = 3). Bars are shown as mean ± SEM (two-way ANOVA (Tukey) *P < 0.05, ns: not significant). **I** Representative real-time cell proliferation curves of BRAFm, mcherry/mcherry β-cateninS33Y OE double transduced NHEMs (left panel) and quantified “slope” (proliferative ability) (right panel) (*n* = 3). Bars are shown as mean ± SEM (two-way ANOVA (Tukey) *P < 0.05, ns: not significant).

As we could show an interaction of YAP and β-catenin in melanoma cell lines, we functionally analyzed the role of YAP and β-catenin in senescence in melanoma. In siYAP treated SBcl2 and WM1366 cells, a significant induction of β-Gal positive cells was detected compared to siCtr (Fig. 7A). MV3 cells showed only a trend towards induction of β-Gal positive cells after the knockdown of YAP compared to control cells. The treatment of SBcl2 and MV3 cells with siβ-catenin led to a significant induction of β-gal positive cells compared to siCtr (Fig. 7B). WM1366 cells showed only a trend towards induction of β-Gal positive cells after the knockdown of β-catenin compared to control cells. The knockdown of YAP had no effects on the number of nuclear PML bodies in SBcl2 and WM1366 cell lines, whereas in MV3 cell line the knockdown led to a statistically significant increased number of nuclear PML bodies compared to control (Fig. 7C). We further measured a significant increased number of nuclear PML bodies after siβ-catenin compared to siCtr in SBcl2, WM1366 and MV3 cells in PML immunofluorescence staining (Fig. 7D). Further, we performed flow cytometry analysis to determine a potential G1 arrest after the knockdown of YAP and β-catenin (Fig. 7E, F). The knockdown of YAP and β-catenin resulted in a strongly increased G1 arrest in SBcl2 and MV3 cell lines compared to control cells. The effects in WM1366 cell lines indicated a trend towards an increase in G1. All analyses in melanoma cell lines regarding the influence of YAP and β-catenin on senescence modulation resulted in the induction of senescent cells caused by treatment with siYAP or siβ-catenin compared to siCtr.

As we demonstrated in Fig. 1, high YAP activity can be observed during OIS in melanocytes. To evaluate if activation of β-catenin may lead to a break of OIS in melanocytes, BRAFm-transduced NHEMs received lentiviral transduction of mutated, constitutively active β-cateninS33Y. This transduction led to an overexpression of β-cateninS33Y in the NHEMs (Supplemental Fig. S1L/M). These double transduced cells (mock/BRAFm and mcherry Ctr/ mcherry β-cateninS33Y) were then analyzed in assays detecting senescence such as SA-β-Gal staining, PML staining, and real-time cell proliferation analysis (RTCA). The SA-β-Gal staining resulted in significantly decreased numbers of β-Gal positive cells in mcherry β-cateninS33Y OE NHEMs compared to corresponding control NHEMs (Fig. 7G). We also measured a strong reduction of PML intensity in β-cateninS33Y OE NHEMs compared to corresponding control NHEMs (Fig. 7H). A central hallmark of cellular senescence is the discontinuation of cell division and thereby proliferation. The overexpression of β-cateninS33Y in NHEMs resulted in an induction of cell growth compared to control NHEMs (Fig. 7I). The analyses in OIS in melanocytes clearly revealed that the overexpression of β-cateninS33Y led to a break of senescence suggesting that interaction of YAP and β-catenin breaks OIS in melanocytes.

### Role of LEF1 in the alternative YAP-β-catenin–signaling axis and its effects on gene expression

**Fig. 8:**
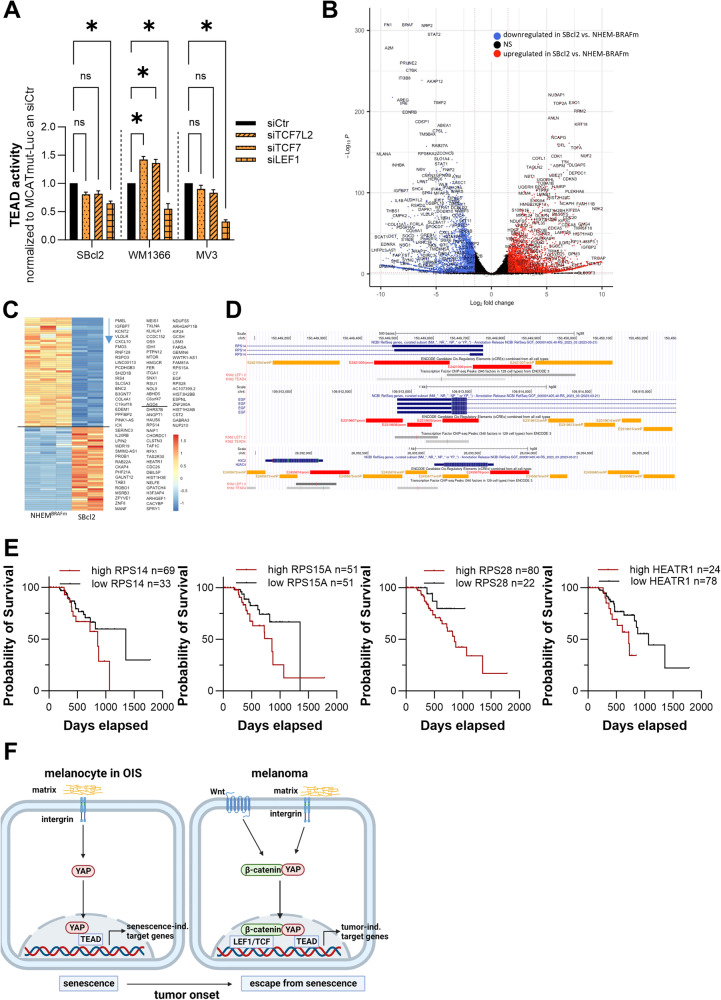
Changes in gene expression pattern due to an alternative YAP-β-catenin signaling axis. **A** Luciferase experiments with MCAT and MCATmut reporter constructs were performed with melanoma cell lines 48 h after siLEF, siTCF7, siTCF7L2 or siCtr transfection. Values were normalized to MCATmut control transfection and siCtr was set as 1 for each cell line (*n* = 3). Bars are shown as mean ± SEM (multiple *T*-tests (Bonferroni-Dunn) *P < 0.05, ns: not significant). **B** The vulcano plot illustrates the differentially expressed genes identified via DESeq2 analysis in SBcl2 cells compared to BRAFm NHEMs (padj < 0.1, -1.5 > LFC > 1.5). **C** The heatmap depicts the differentially expressed genes in SBcl2 cells compared to BRAFm NHEMs containing a TEAD and LEF binding site (motif) around their TSS. **D** Illustration of the combinational TEAD and LEF binding sites around the TSS of the differentially expressed genes RPS14, EGF, and H2A using publicly available ChIP-seq tracks for LEF1 and TEAD4 from K562 cells. Visualization of the tracks was made using the UCSC genome browser (https://genome.ucsc.edu/). **E** Survival analysis in a skin cutaneous melanoma (SKCM) patient data set comparing low and high RPS14, RPS15A, RPS28, HEATR1, and FARSA levels. **F** Schematic summary of the main results (created with BioRender).

Since we observed an alternative YAP-β-catenin signaling axis that disrupted OIS in melanocytes, we were interested in how the interaction of YAP-β-catenin leads to the switch from senescence-stabilization to a tumor-promoting phenotype. Members of the TCF/LEF transcription factor family contain an N-terminal domain responsible for DNA-binding and a C-terminal β-catenin-binding domain. Since β-catenin can directly bind to the TCF/LEF1 transactivation domain, we investigated whether the TCF/LEF1-TEAD interaction is required for this alternative YAP-β-catenin signaling axis. To test this possibility, we transfected SBcl2, WM1366, and MV3 cells with siRNA pools specific for TCF7, TCF7L2, or LEF1, respectively. Transfection of melanoma cell lines led to a reduction in TCF7, TCF7L2, and LEF1 mRNA expression of ~50–80% after 48 h (Supplemental Fig. S1N/O/P). Additionally, siTCF7, siTCF7L2 and LEF1 reduced protein expression by ~60–80% after 48 h (Supplemental Fig. S1Q/R/S; uncropped blots and molecular weight markers are shown in Supplemental Fig. S6/7). MCAT luciferase reporter assays performed after siRNA pool transfection revealed significantly reduced TEAD reporter activity due to siLEF1 compared to siCtr in all melanoma cell lines (Fig. 8A). In SBcl2 and MV3 cells, knockdown of TCF7 and TCF7L2 led to a reduction in TEAD reporter activity compared to that in control cells. Only in the WM1366 cell line did the transfection of TCF7 and TCF7L2 result in an increase in TEAD reporter activity compared to that in the siCtr group. These observations suggest that the interaction between TCF/LEF1 and TEAD could play a role in the YAP-β-catenin-mediated shift in gene expression. As the knockdown of LEF1 resulted in major effects on MCAT reporter activity, we focused on that transcription factor in further experiments. First, we performed differential expression analysis based on RNA sequencing data of the melanoma cell line SBcl2 compared to BRAFm-transduced NHEMs, comparing a melanoma cell line generated from an early in situ melanoma to senescent melanocytes and focusing thereby on the step of disrupting OIS. Interestingly, we observed large differences in gene expression between the compared cellular systems. A total of 3968 genes were shown to be significantly upregulated, and 4416 were significantly downregulated in SBcl2 melanoma cells compared to BRAFm-transduced NHEMs (padj < 0.1, -1.5 > LFC > 1.5) (Fig. 8B). We subsequently investigated which of the identified differentially expressed genes (DEGs) might be regulated by the combined binding of the transcription factors YAP and β-catenin. Therefore, we performed a transcription start site (TSS)-centered genome-wide motif analysis of TEAD and LEF1 binding sites. Indeed, we detected a subset of the identified differentially regulated genes with motifs for both transcription factors in close proximity to their TSS (Fig. 8C). Supplemental Table 1 illustrates the 95 DEGs that have both a TEAD and a LEF1 binding site around their TSS with a maximum spacing of 100 bp. Figure 8D exemplarily illustrates the co-appearance of LEF1 and TEAD binding sites in CIS regulatory elements of the respective differentially expressed genes (RPS14, EGF, H2AC4; https://genome.ucsc.edu).

These results suggest that the YAP-β-catenin complex may act as an enhancer and silencer during tumor development but is not required during the induction of OIS.

Functional annotation analysis of the potentially YAP-β-catenin-regulated genes using the *Database for Annotation, Visualization and Integrated Discovery* (DAVID) resulted in 7 clusters. Cluster generation was performed by focusing on Gene Ontology (GO) terms and signaling pathways (Wiki, REACTOME and KEGG pathways).

Interestingly, the clustered genes were involved mainly in rRNA processing (RPS14, RPS28, RPS15A, HEATR1, and NOL9), ribosomal biogenesis (RPS14, RPS28, and RPS15A), and cell cycle processes (NUP210, CDC26, HAUS6, and LPIN2).

Analysis of the human protein atlas revealed that patients with high protein expression had shorter survival than patients with low protein expression (Fig. 8E).

We therefore concluded that the gene expression of these cells changes via an alternative YAP-β-catenin signaling axis and the interaction of the transcription factors LEF1 and TEAD, which leads to a disruption of the OIS in NHEMs (Fig. 8F).

## Discussion

In this study, we revealed that the induction of Wnt ligand expression is involved in early melanoma development. The Wnt pathway is known to play a crucial role in embryonic development and stem cell proliferation [56]. Here, the importance of the oncogenic potential of defined Wnt ligands was demonstrated in early events of melanoma development.

Incubation with just one Wnt ligand, either Wnt6 or Wnt10b, led to a disruption in the OIS of BRAFm-transduced melanocytes from OIS, whereas in melanoma cells, knockdown of the respective Wnt6 expression induced a senescent phenotype. Consistent with these findings, our results revealed that treatment with AMBMP, a Wnt agonist, resulted in a disruption of the OIS.

Indicating the wide range of functional effects of Wnts not only in early tumor development, data analysis of RNA samples obtained from melanoma patients with positive sentinel lymph nodes (SLNs) revealed that Wnt10b is associated with melanoma recurrence in older patients with tumor-positive SLNs [57]. Furthermore, recent studies have shown the role of the Wnt pathway in aging organisms and that this pathway may affect age-related melanoma outcomes through several different mechanisms [19]. In contrast, Ye et al. showed that Wnt10b promotes an increase in the number of mature melanocytes and their pigmentation, suggesting that Wnt10b promotes melanocyte differentiation of mouse hair follicle melanocytes [58]. Another study described that co-culture of B16 cells with conditioned medium from Wnt10b-producing COS cells resulted in reduced proliferation and senescence induction [59]. The differences in our results are probably due to differences in the experimental designs. Ye et al. used a mouse melanocyte cell line, which differs from our use of neonatal primary melanocytes. A study of B16 cells did not reveal the expression levels of Wnt10b. Our melanoma cell lines showed high Wnt10b mRNA expression, which indicates that these cell lines differ in terms of their mRNA expression patterns, which led to different effects.

No data are available on Wnt6 in melanoma, but in other tumor types, Wnt6 has been described to act as an oncogene [60–62].

Furthermore, Weeraratna et al. reported that Wnt5a plays a major role in melanoma by directly modulating the motility and invasion of metastatic melanoma cells and promoting metastasis via a β-catenin-independent mechanism [20, 63]. Interestingly, compared with NHEMs, all our melanoma cell lines tended to exhibit reduced expression of Wnt5a. In support of our data, Pham et al. and Forno et al. reported high Wnt5a expression in nevi [63, 64]. These results indicate that Wnt5a is more involved in the later stages of melanoma development, resulting in tumor dissemination, but not in early processes. Furthermore, Wnt1 and Wnt3a play critical roles in the development of the neural crest. Wnt1 and Wnt3a also promote the development of neural crest cells into pigmented cells by increasing the number of melanocytes or specifying to melanocytes. Interestingly, in melanoma, the induction of Wnt3a led to reduced proliferation in vivo and decreased tumor size due to the activation of Wnt/β-catenin [65]. These results indicate that the tumor suppressive function of Wnt3a is due to classical β-catenin activity, which was not active in the melanoma cells used in this study and was therefore not of interest. In support of the suggestion that Wnt ligands might also have tumor suppressive functions through various context-dependent pathways, analysis of the transcriptomic profiles of Wnt ligands across multiple tumor types revealed a tissue-dependent and tumor-dependent pattern of Wnt ligand expression. Chen et al. suggested that Wnt3 and Wnt4 are tumor suppressors in melanoma [66]. There are even more Wnt ligands, such as Wnt2 and Wnt7a, that have been described to have tumor suppressive effects on different cancer types and on melanoma cells (Wnt2) [67, 68]. Interestingly, our data analysis did not reveal Wnt2 upregulation in primary tumors or metastases. In support of our hypotheses that Wnt ligands might have a compensatory function and that the combination of multiple Wnt ligands would result in stronger effects, Cha et al. and Miller et al. demonstrated that Wnt5a/Wnt11 or Wnt2/Wnt7b cooperate and induce Wnt signaling [69, 70].

Studies by Ozhan et al. in zebrafish showed that Wnt pathway-related genes are differentially regulated between regenerating melanocytes and melanoma cells. While activation of the canonical β-catenin signaling pathway promoted regeneration of melanocytes, activation in melanoma cells led to suppression of invasiveness, migration and proliferation [71]. The molecular mechanisms by which β-catenin induced the functional effects were not elucidated. However, Shakhova et al. were able to show that the protein level of SOX10 was reduced by the activation of β-catenin, which also led to a reduction in proliferation and invasion in melanoma cell lines [72]. Supporting our data, Wang et al. were able to show that activation of the β-catenin signaling pathway has a tumor-promoting effect via a further molecular pathway [73]. In summary, the activation of β-catenin signaling appears to be influenced by many factors and activates tumor-promoting and tumor-protective processes due to various interaction partners [69, 70].

Following the induction of Wnt6 and Wnt10b expression, the activation of β-catenin leads to the disruption of OIS in BRAFm-transduced melanocytes, as demonstrated in our study, most impressively after the use of constitutively active β-catenin. Furthermore, we demonstrated that this canonical Wnt pathway was activated in melanoma cells due to the upregulation of Wnt ligands. Similarly, another study showed that 30% of melanoma biopsies harbor β-catenin in their nuclei, indicating an active Wnt/β-catenin pathway [23]. Interestingly, the melanoma cells analyzed in this study did not exhibit classical β-catenin activity, as measured by a luciferase reporter assay. This indicated that the activation of β-catenin in melanoma cells resulted in an alternative signaling pathway to overcome OIS in melanocytes. Supporting our data, in transgenic mice expressing an activated form of β-catenin in NRAS-mutated melanocytes, active β-catenin leads to immortalization of melanocytes by suppressing p16^INK4a^ gene expression [24].

Furthermore, we observed activation of YAP in BRAFm-transduced melanocytes. With respect to the BRAFm-transduced NHEMs, we measured the mRNA levels of YAP and classical YAP targets, such as AXL, AREG and CTGF, indicating nuclear YAP activity in OIS. Immunofluorescence staining of the YAP protein confirmed the induction of nuclear YAP localization in BRAFm-transduced NHEMs in OIS cells compared to that in control cells. A recently published study revealed that BRAF^V600E^-expressing melanocytes in nevi exhibit decreased YAP/TAZ signaling via phosphorylation of YAP by the hippo kinase Lats1/2 (activation of the Hippo pathway), thereby restraining oncogenic melanocyte proliferation [74]. The differences in our results are probably due to differences in the experimental designs. The mouse data represent YAP/TAZ activity in hair follicle melanocytes. While epidermal melanocytes, which were used in this study, are differentiated, hair follicle melanocytes harbor different differentiation states, depending on the hair follicle stage [75]. Nevertheless, Vittoria et al. observed clusters in which YAP/TAZ activity was much greater than that in other clusters, suggesting that not only a cell-intrinsic mechanism following BRAF^V600E^ expression led to reduced YAP/TAZ activity but also that the cell-extrinsic mechanism might play a role [74]. Cell culture experiments were performed with either immortalized melanocytes or adult primary melanocytes and with different overexpression systems at different timepoints. Both cell types differ from our used neonatal primary melanocytes, and timepoints play an important role in the analysis of tumor development. However, the role that YAP plays in maintaining OIS was not the topic of this study; therefore, no further experiments were performed.

Interestingly, many studies of different tumor types have shown that activation of YAP is positively correlated with malignancy, relapse, metastasis, chemoresistance and decreased overall survival [10, 76]. Additionally, in melanoma, YAP is an oncoprotein that promotes melanoma progression, cell invasion, metastasis and a poor prognosis [77, 78], supporting our finding that YAP is still relevant for preventing OIS, but we revealed that it is not an inducer of this process. To understand the underlying mechanism, we analyzed the association of YAP with different transcription factors, first focusing on PAX3 and TBX5. With a luciferase reporter, we could not confirm that YAP interacted with PAX3 or TBX5, suggesting that the microenvironment and other transcription factors, as binding partners, are important. In contrast, Miskolczi et al. reported that nuclear YAP/TAZ expression increases with increased collagen abundance in melanoma and that YAP interacts with PAX3 and induces microphthalmia-associated transcription factor (MITF) expression at the mRNA and protein levels [79]. This mechanism could depend on the microenvironment, as more collagen is relevant here, suggesting further regulatory aspects.

As described above, the overexpression of the Wnt ligands Wnt6 and Wnt10b and the activation of β-catenin led to a disruption of OIS in BRAFm-transduced melanocytes, suggesting that β-catenin functions as a YAP-interacting protein. In further experiments, we showed that in melanoma cells, YAP and β-catenin interact with each other at the DNA and confirmed the presence of an alternative YAP-β-catenin signaling axis without activating classical β-catenin targets (such as TOPflash). In support of our data, Azzolin et al. revealed an interaction between YAP and β-catenin in HEK293T cells, showing that YAP/TAZ is directly associated with β-catenin. Upregulation of the YAP protein effectively inhibited classical canonical Wnt signaling even during treatment with recombinant Wnt3a [25]. Another group demonstrated that YAP and phosphorylated YAP can bind directly to β-catenin and inhibit TOP flashing activity [26].

Therefore, we expected a change in the gene expression pattern from senescence-stabilized genes to tumor-supportive genes via the β-catenin/YAP interaction. Experiments with siLEF1, siTCF7 or siTCF7L2 resulted in significantly reduced YAP/TEAD activity compared to that in control cells. Thus, we speculated that the observed TCF/LEF1-TEAD interaction is required for this alternative YAP-β-catenin signaling axis. No data are available on the TCF/LEF1-TEAD interaction in melanoma. In support of our data, in mouse cardiomyocytes, YAP and β-catenin are recruited to common target genes (including *Sox2* and *Snai2*) through the TEAD and TCF transcription factors, respectively, and cooperatively activate these genes [80]. Another example of the functional interplay of the TCF and TEAD transcription factors has been described in colorectal carcinoma [81].

Expression analysis of the melanoma cell line SBcl2 compared to BRAFm-transduced NHEMs revealed that both transcription factor motifs were in close proximity to the TSS in the promoters of the differentially regulated genes. Functional annotation analysis of the potentially YAP/β-catenin-regulated genes resulted in 7 clusters, one of which was associated with rRNA processing. Ribosomal proteins such as RPS14, PRS15A, RPS28, HEAT repeat containing 1 (HEATR1) and phenylanlanyl-TRNA synthetase subunit alpha (FARSA) were deregulated due to co-binding of YAP and β-catenin to LEF1/TEAD. Therefore, we suggest that an alternative YAP-β-catenin signaling axis leads to the overexpression of ribosomal proteins, which causes a disruption of OIS and tumor progression. RPS14, RPS15a and RPS28 encode proteins that are components of the small 40 S subunit, and together with the large 60 S subunit, they catalyze protein synthesis. Tumor cells are characterized by increased production of ribosomes, which are necessary to sustain enhanced growth and subsequent cell division. Interestingly, recent studies have identified a link between abnormal ribosome biogenesis and increased tumor burden [82–84]. RPS14 overexpression promoted the development of colorectal carcinoma, and RPS14, a downstream target of zinc finger protein 280 A (ZNF280A), was upregulated in melanoma cells and has a co-binding site for YAP and β-catenin via LEF1/TEAD [85]. These findings strongly support our finding that YAP/β-catenin co-binding induces early tumor development. In addition to our findings, Bowley et al. identified a common melanoma-related circulating tumor cell gene signature for ribosomal protein large/small subunits (RPL/RPS) that is associated with the onset and progression of melanoma brain metastasis [86]. In hepatocellular carcinoma, RPS15A is upregulated, and high RPS15A expression predicts poor survival. The finding that RPS15A knockdown inhibits angiogenesis and tumor growth is also in line with our hypotheses that the overexpression of ribosomal proteins causes OIS disruption and tumor progression [87].

In summary, this study showed that overexpression of Wnt and activation of β-catenin lead to proliferation and inhibition of senescence in BRAFm-transduced melanocytes via an alternative YAP-β-catenin signaling axis. Analysis of the expression of Wnt6 or Wnt10b in BRAFm-mutated melanocytes could reveal that these genes could serve as biomarkers for early malignant transformation. Therapeutic intervention targeting the alternative YAP-β-catenin signaling axis and therefore promoting reinduction of senescence in melanoma cells could be a new opportunity for cancer therapy. The expression of Wnt ligands could serve as biomarker not only for early malignant transformation, but also as a prognostic factor for the recurrence of tumors in older patients. In further studies, we would like to investigate whether more Wnt ligands have prognostic potential, if Wnt ligands can also have a tumor-preventive effect and whether a combination of several Wnt ligands can achieve even stronger effects. The analyses may lead to a signature for a better assessment of the individual prognosis of patients and thus enables specific and effective therapy. Furthermore, patients with a critical Wnt ligand signature could be monitored more closely in order to detect a recurrence of the tumor at an early stage. Finally, the possible role of YAP as a OIS inducer in melanocytes might exhibit further details of the underlying signaling network and might give implications for the prognosis of additional tumor entities.

### Supplementary information

Suppl. Figures

original data

AJ-Checklist

SupplTable 1, 2

## Data Availability

The data generated in this study are available within the article and its supplemental data files. Further, the data analyzed in this study were obtained from Gene Expression Omnibus (GEO) at GSE55709, GSE64758, and GSE37350.
