# Supplementary material for: Alternative Wnt-signaling axis leads to a break of oncogene-induced senescence
Source: Cell Death Dis. 2024 Feb 22;15(2):166. doi: 10.1038/s41419-024-06550-8 (PMC10883971; doi:10.1038/s41419-024-06550-8)
Supplement: Supplementary file 1 — Suppl. Figures [file 41419_2024_6550_MOESM1_ESM.pdf]

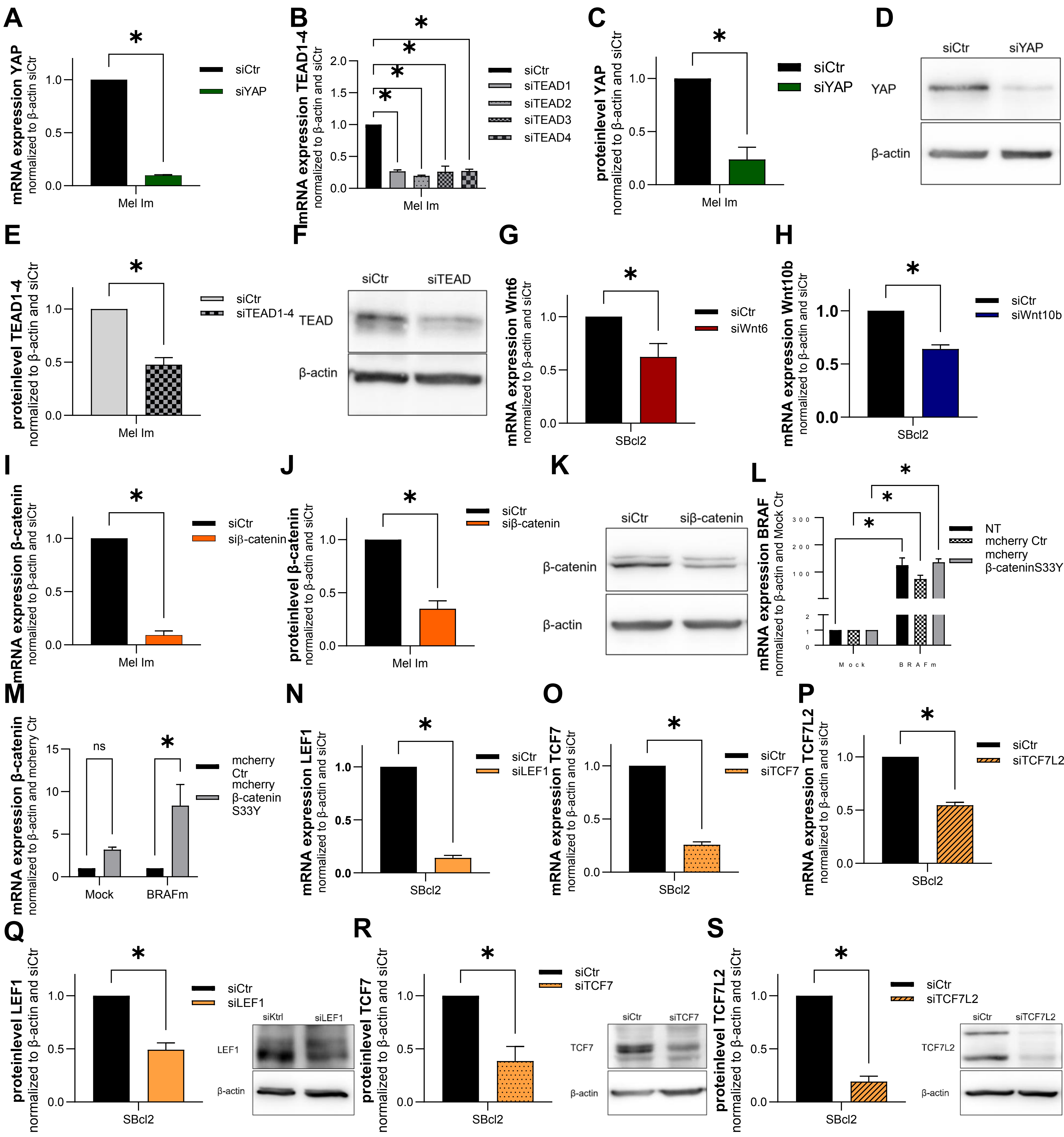

Figure S1: Validation siRNA pool

A: Relative YAP mRNA expression in Mel Im treated with siYAP and siCtr for 48 h measured by qRT-PCR. mRNA level in siCtr is set to 1. **B:** Relative TEAD1-4 mRNA expression in Mel Im treated with siTAD1-4 and siCtr for 48 h measured by qRT-PCR. mRNA level in siCtr is set to 1. **C/D:** Western Blot analysis of YAP protein expression in Mel Im treated with siYAP and siCtr for 48 h. Protein level in siCtr is set to 1. **E/F:** Western Blot analysis of TAD1-4 protein expression in Mel Im treated with siTEAD1-4 and siCtr for 48 h. Protein level in siCtr is set to 1. **G:** Relative Wnt6 mRNA expression in SBcl2 treated with siWnt6 and siCtr for 48 h measured by qRT-PCR. mRNA level in siCtr is set to 1. **H:** Relative Wnt10b mRNA expression in SBcl2 treated with siWnt10b and siCtr for 48 h measured by qRT-PCR. mRNA level in siCtr is set to 1. **I:** Relative β-catenin mRNA expression in Mel Im treated with siβ-catenin and siCtr for 48 h measured by qRT-PCR. mRNA level in siCtr is set to 1. **J/K:** Western Blot analysis of β-catenin protein expression in Mel Im treated with siβ-catenin and siCtr for 48 h. Protein level in siCtr is set to 1. **L:** Relative BRAF mRNA expression in mock/BRAFm NHEM seven days after transduktion measured by qRT-PCR. mRNA level in moch is set to 1. **M:** Relative β-catenin mRNA expression in mock/BRAFm and mcherry Ctr/ mcherry β-cateninS33Y seven days after transduktion measured by qRT-PCR. mRNA level in moch is set to 1. **N:** Relative LEF1 mRNA expression in SBcl2 treated with siLEF1 and siCtr for 48 h measured by qRT-PCR. mRNA level in siCtr is set to 1. **O:** : Relative TCF7 mRNA expression in SBcl2 treated with siTCF7 and siCtr for 48 h measured by qRT-PCR. mRNA level in siCtr is set to 1. **P:** : Relative TCF7L2 mRNA expression in SBcl2 treated with siTCF7L2 and siCtr for 48 h measured by qRT-PCR. mRNA level in siCtr is set to 1. **Q:** Western Blot analysis of LEF1 protein expression in SBcl2 treated with siLEF1 and siCtr for 48 h. Protein level in siCtr is set to 1. **R:** Western Blot analysis of TCF7 protein expression in SBcl2 treated with siTCF7 and siCtr for 48 h. Protein level in siCtr is set to 1. **S:** Western Blot analysis of TCF7L2 protein expression in SBcl2 treated with siTCF7L2 and siCtr for 48 h. Protein level in siCtr is set to 1. Values represent the mean ± SEM of 3 independent experiments (students T-test *$P < 0.05$, ns: not significant).

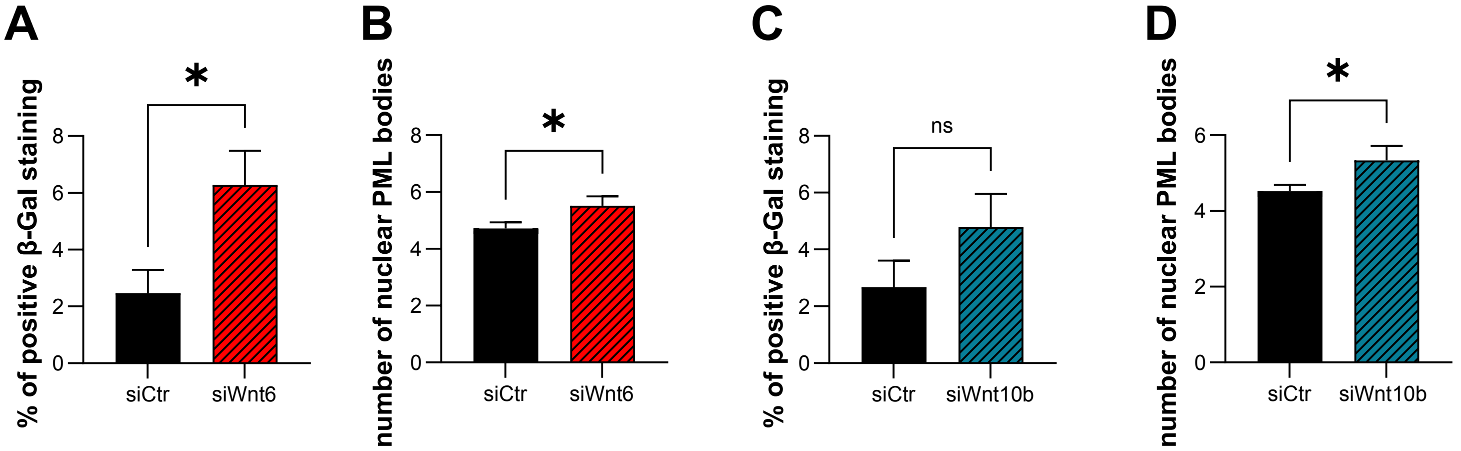

Figure S2: Role of Wnt6 and Wnt10b in BRAF mutated melanoma cells

A: Percentages of SA-β-Galactosidase positive cells in Mel Im cell line 48 h after transfection with siWnt6 or siCtr. **B:** Immunofluorescence staining of PML and DAPI in Mel Im cell line 48 h after the transfection with siWnt6 or siCtr. The graph shows the number of nuclear PML bodies. **C:** Percentages of SA-β-Galactosidase positive cells in Mel Im cell line 48 h after transfection with siWnt10b or siCtr **D: :** Immunofluorescence staining of PML and DAPI in Mel Im cell line 48 h after the transfection with siWnt10b or siCtr. The graph shows the number of nuclear PML bodies. Values represent the mean ± SEM of 3 independent experiments (students T-test *P < 0.05, ns: not significant).

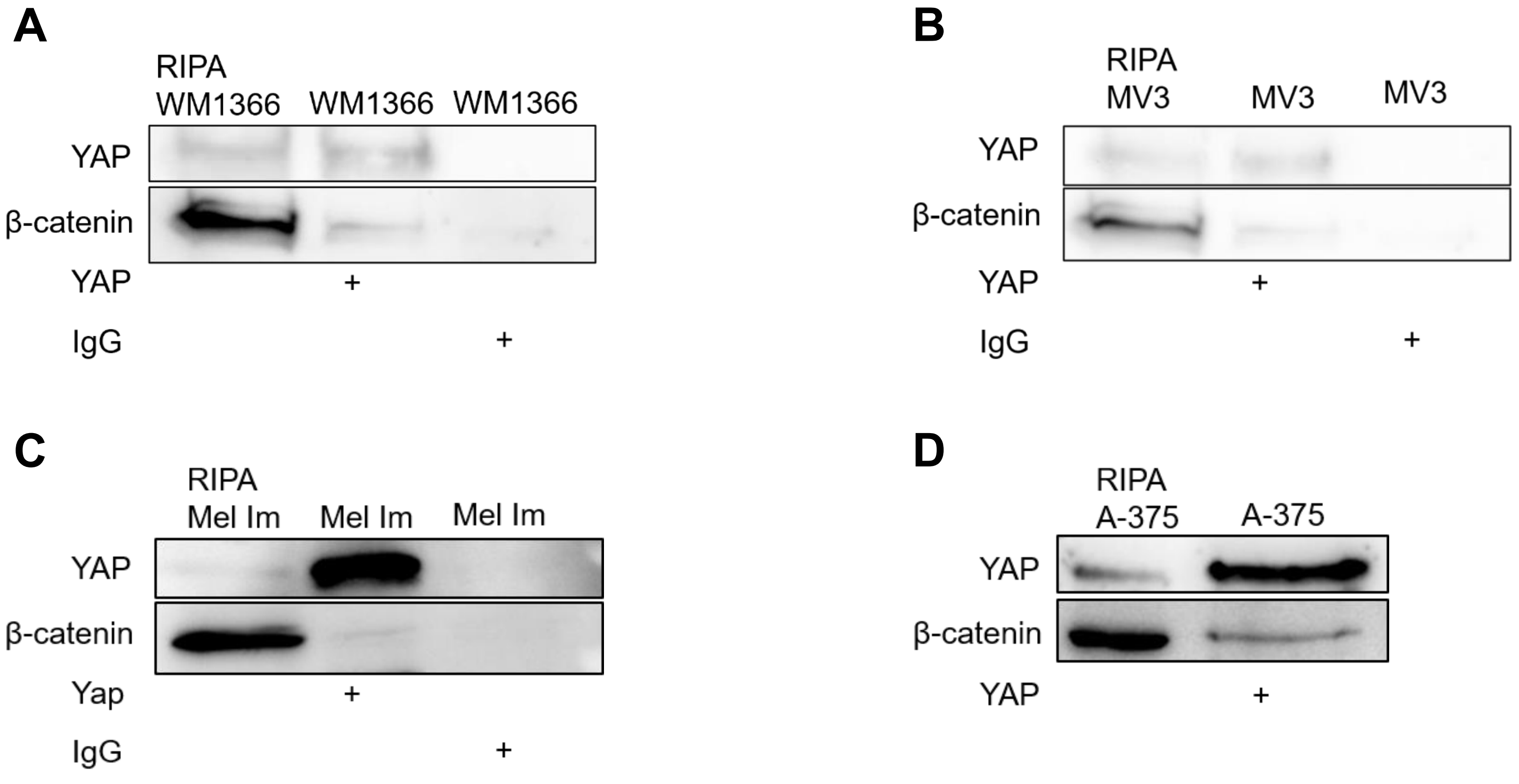

Figure S3: Co-immunoprecipitation

A: Interaction of YAP and β-catenin measured by co-immunoprecipitation with anti-YAP antibody in WM1366 cells (lane 2). Lane 1 represents input of WM1366 cells and lane 3 immunoprecipitation with anti-IgG antibody in WM1366 cells. Immunoblotting with anti-YAP and anti β-catenin antibodies. **B:** Interaction of YAP and β-catenin measured by co-immunoprecipitation with anti-YAP antibody in MV3 cells (lane 2). Lane 1 represents input of MV3 cells and lane 3 immunoprecipitation with anti-IgG antibody in WM1366 cells. Immunoblotting with anti-YAP and anti β-catenin antibodies. **C:** Interaction of YAP and β-catenin measured by co-immunoprecipitation with anti-YAP antibody in Mel Im cells (lane 2). Lane 1 represents input of Mel Im cells and lane 3 immunoprecipitation with anti-IgG antibody in Mel Im cells. Immunoblotting with anti-YAP and anti β-catenin antibodies. **D:** Interaction of YAP and β-catenin measured by co-immunoprecipitation with anti-YAP antibody in A-375 cells (lane 2). Lane 1 represents input of A-375 cells. Immunoblotting with anti-YAP and anti β-catenin antibodies.
