# Supplementary material for: Alternative Wnt-signaling axis leads to a break of oncogene-induced senescence
Source: Cell Death Dis. 2024 Feb 22;15(2):166. doi: 10.1038/s41419-024-06550-8 (PMC10883971; doi:10.1038/s41419-024-06550-8)

Figure S4: uncropped blots

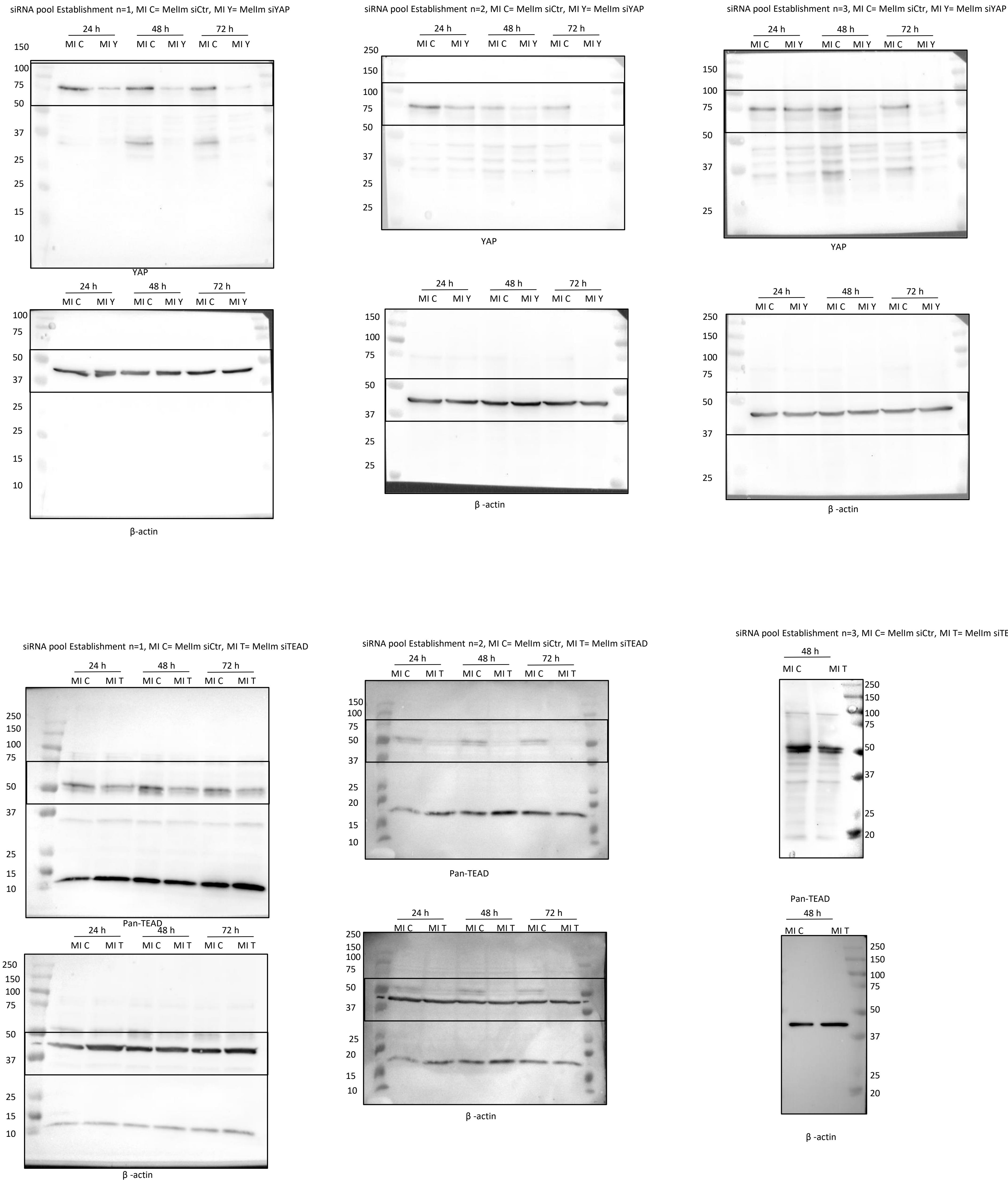

Figure S5: uncropped blots

siRNA pool Establishment n=1, MI C= Mel Im siCtr, MI β= Mel Im si β-catenin

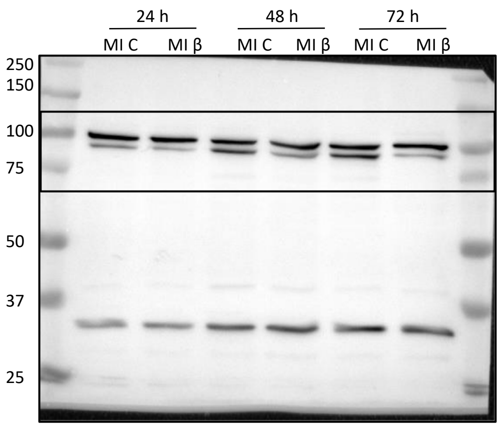

β-catenin

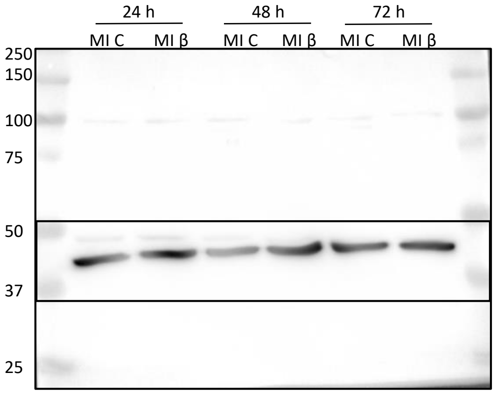

β-actin

siRNA pool Establishment n=2, MI C= Mel Im siCtr, MI ß= Mel Im si β-catenin

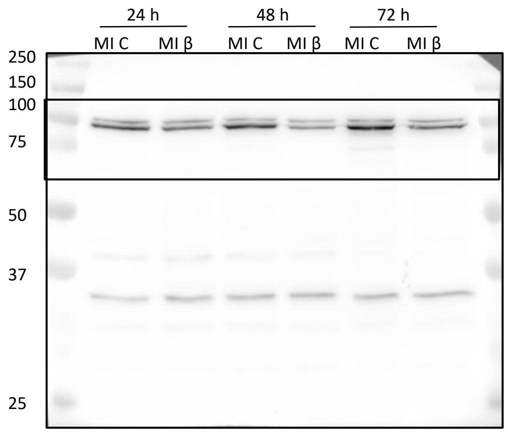

β-catenin

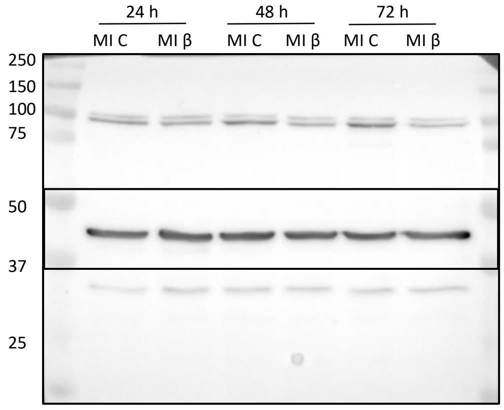

β-actin

siRNA pool Establishment n=3, MI C= Mel Im siCtr, MI β= MelIm si β-catenin

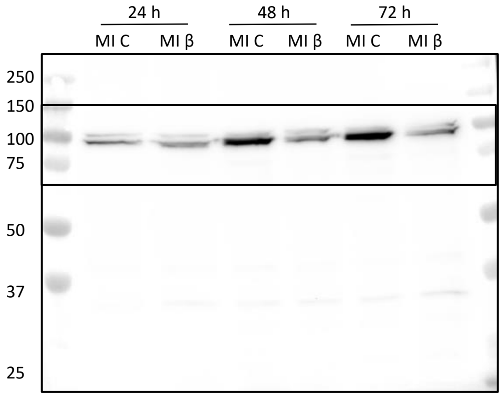

β-catenin

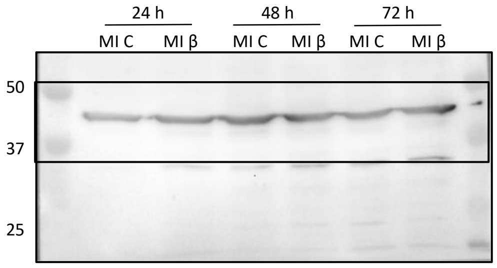

β-actin

siRNA pool Establishment n=5 SB C= SBcl2 siCtr, SB β = SBcl2 si β-catenin

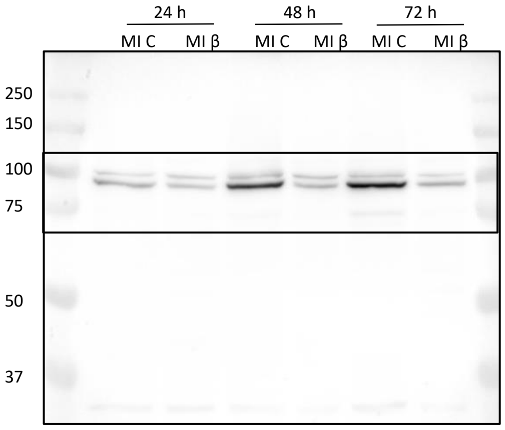

β-catenin

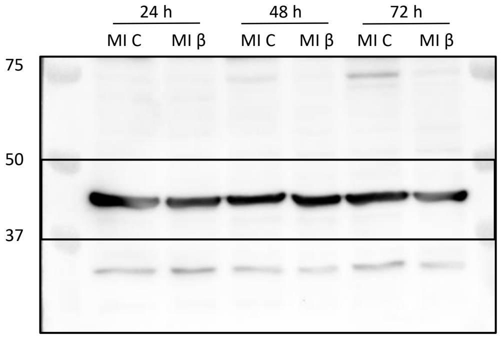

β-actin

Figure S6: uncropped blots

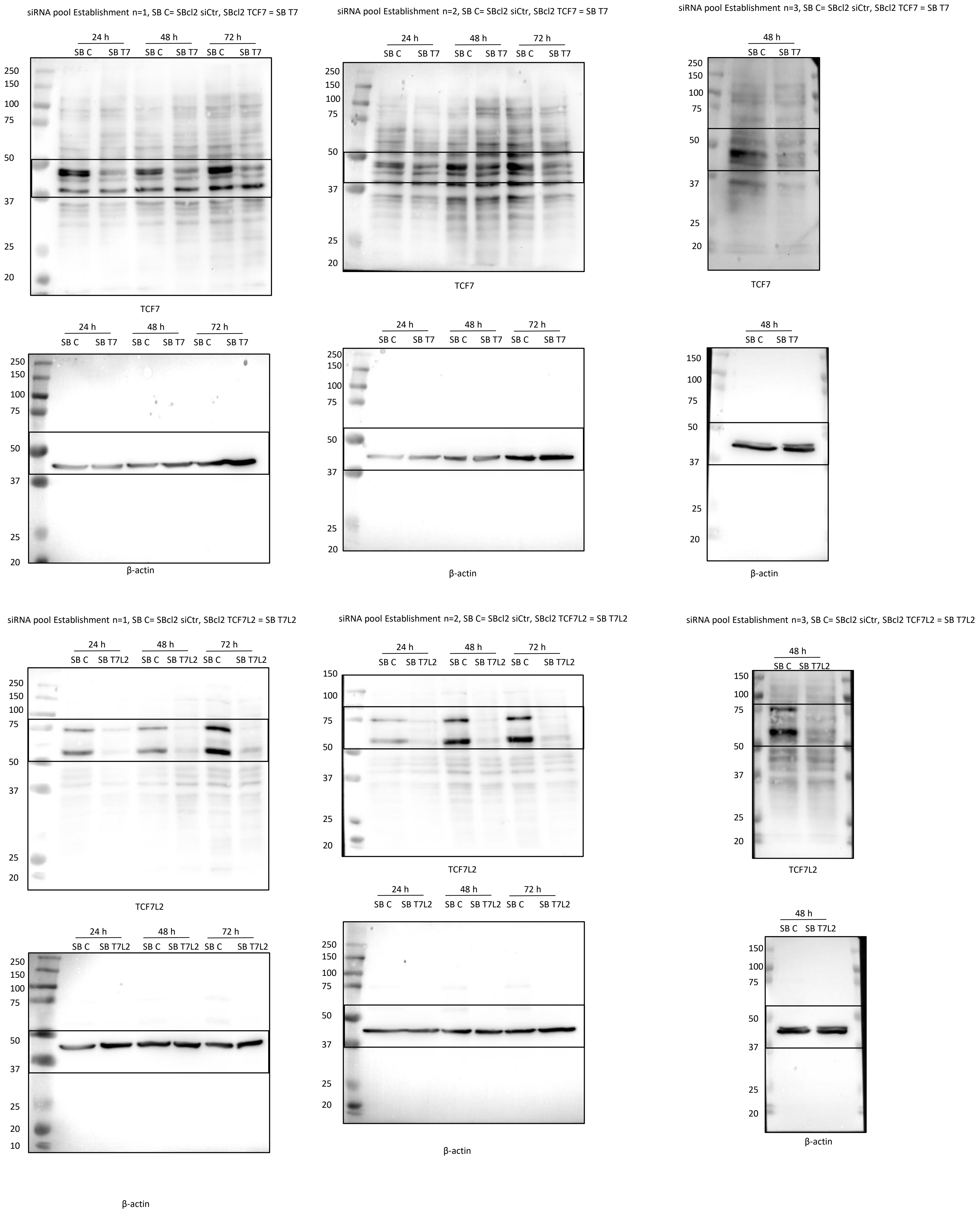

Figure S7: uncropped blots

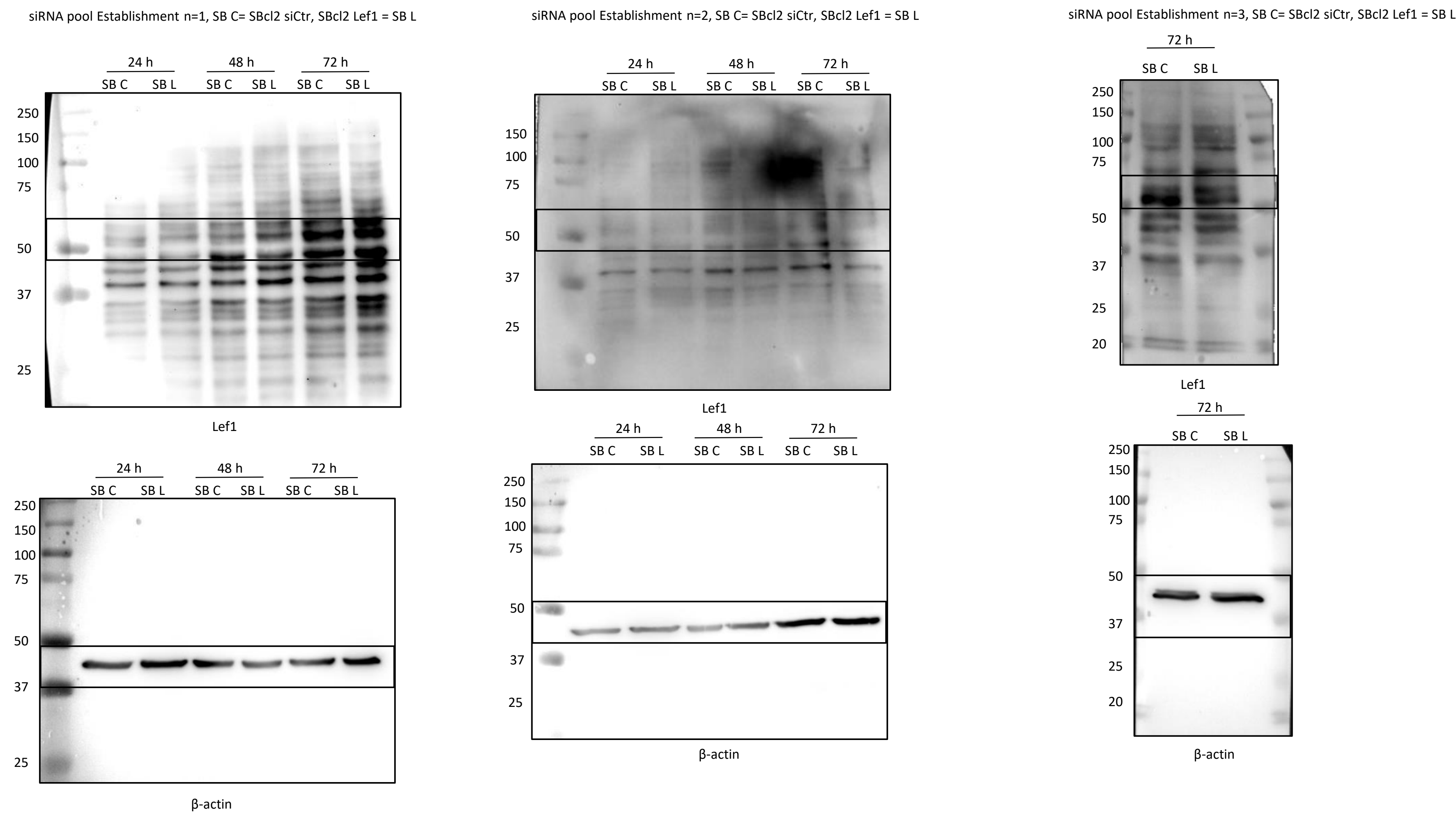

Supplement: Supplementary file 2 — original data [file 41419_2024_6550_MOESM2_ESM.pdf]
